# Novel drug-inducible CRISPRa/i systems for rapid and reversible manipulation of gene transcription

**DOI:** 10.1007/s00018-025-05786-7

**Published:** 2025-06-23

**Authors:** Ming Sui, Meiling Zhou, Mengge Cui, Huan Liu, Xiaolin Zhang, Na Hu, Yang Li, Beibei Wang, Guojun Yang, Pengling Gui, Lingqiang Zhu, Feng Wan, Bin Zhang

**Affiliations:** 1 Department of Physiology, School of Basic Medicine, Tongji Medical College, Huazhong University of Science and Technology, Wuhan, 430030 China; 2 The Institute for Brain Research, Collaborative Innovation Center for Brain Science, Huazhong University of Science and Technology, Wuhan, 430030 China; 3 Hubei Key Laboratory of Drug Target Research and Pharmacodynamic Evaluation, Huazhong University of Science and Technology, Wuhan, 430030 China; 4 Key Laboratory of Hubei Province for Cognitive and Affective Disorders, School of Medicine, Wuhan Institute of Biomedical Sciences, Jianghan University, Wuhan, 430056 China; 5 Department of Clinical Laboratory, The 980th Hospital of PLA Joint Logistical Support Force (Bethune International Peace Hospital), Shijiazhuang, 050000 China; 6 Department of Neurosurgery, Guangdong Provincial People’s Hospital (Guangdong Academy of Medical Sciences), Southern Medical University, Guangzhou, 510080 China; 7 Department of Blood Transfusion, Zhongnan Hospital of Wuhan University, Wuhan, 430071 China; 8 School of Medicine, Hebei University of Engineering, Handan, 056038 China; 9 Department of Pathophysiology, School of Basic Medicine, Tongji Medical College, Huazhong University of Science and Technology, Wuhan, 430030 China

**Keywords:** iCRISPRa/i, 4OHT, Transcriptional regulation, Phenotypic change

## Abstract

CRISPR activation and interference (CRISPRa/i) are highly effective tools to regulate transcription by fusing dead Cas9 (dCas9) with transcriptional regulatory factors guided by small guide RNA (sgRNA) in mammalian cells and mice. Still, a controllable gene regulation system is desired to investigate and manipulate dynamic biological processes. Here, we reported flexible drug-responsive CRISPRa/i systems by fusing mutated human estrogen receptor (ERT2) domains, which responded to estrogen analogue tamoxifen or its active metabolite 4-hydroxy-tamoxifen (4OHT), to CRISPRa/i components for transcriptional regulation. Upon 4OHT treatment, the optimal variants, ERT2-ERT2-CRISPRa/i-ERT2 (iCRISPRa/i), showed rapid protein translocation of iCRISPRa/i from cytoplasm to nucleus and subsequent transcriptional response. The inducible transcriptional manipulation could be restored to its original level when 4OHT was withdrawn. Moreover, the efficiencies of gene expression regulation of iCRISPRa/i were comparable to those of non-inducible and doxycycline-inducible counterparts, with a lower leakage and a faster drug response activity. The iCRISPRa/i systems successfully induced phenotypic changes in various cell lines. These results highlight that iCRISPRa/i systems could achieve fast and flexible drug-responsive transcriptional modulation and phenotypic changes, and thus provide better options for gain- and loss-of-function model construction and gene therapy.

**Supplementary Information:**

The online version contains supplementary material available at 10.1007/s00018-025-05786-7.

## Introduction

The regulation of gene transcription is involved in the whole process of life, such as growth, development and death, the emergence of CRISPR (clustered regularly interspaced short palindromic repeats)/Cas9 system made gene expression manipulation simple, rapid and convenient. Catalytically inactive dead Cas9 (dCas9) that co-expressed with a sgRNA could generate a DNA recognition complex and repress gene expression by specifically interfering with transcriptional elongation, RNA polymerase binding, or transcription factor binding without cutting DNA which is known as CRISPR interference (CRISPRi) [1]. Further study showed that dCas9 could be used as a more efficient CRISPRi platform when fused with a transcription suppressor, the KRAB (Krüppel-associated box) domain of Kox1 [2]. Moreover, the direct domain fusion strategy could also generate CRISPR activation (CRISPRa) systems, such as dCas9-VP64 [3, 4] and dCas9-VPR (VP64-p65-Rta) [5].

One characteristic of the CRISPR activation and interference (CRISPRa/i) systems is their constitutive expression and continuous transcriptional manipulation in the host cells, which restricts their application for temporally precise regulation of target genes for the following reasons. Firstly, the downregulation of critical genes essential for embryo development could be risky in transgenic animals because it would induce embryonic lethality [6, 7]. Next, prolonged expression of the sgRNA and dCas9 can increase the potential for off-target effects [8]. Last but not least, gene expression regulation achieved by non-inducible CRISPRa/i could not acquire the ideal precision and flexibility. To overcome these disadvantages, several inducible CRISPRa/i systems have been developed for cell, tissue, or organ-specific gene manipulation in a particular developmental stage, with advantages over conventional CRISPRa/i systems, including decreased toxicity for long survival and precise transcriptional manipulation for faithful/accurate modeling of disease pathogenesis. Previous designs of these spatially and temporally controllable systems include drug- [9–11], light- [12–15], chemical- [16] and heat-shock- [17] inducible systems. Although these approaches could achieve inducible gene expression regulation, they could not manipulate gene expression in a simple, rapid, controllable and reversible manner and achieve expected phenotypic changes in mammalian cells, thus a superior method is necessary to fulfill these requirements.

Recently, a fusion protein composed of Cas9 endonuclease and a mutant form of the ligand binding domain of the estrogen receptor (ERT2) has been developed, its genome editing activity can be switched on and off in human cells [18]. The ERT2 domain causes the fusion protein to be sequestrated in the cytoplasm by interacting with HSP90, thereby preventing the fusion protein from translocating to the nucleus [19, 20]. Once induced by tamoxifen or its metabolites, 4-hydroxy-tamoxifen (4OHT) or 4-hydroxy-N-desmethyltamoxifen (endoxifen), the fusion protein will be dissociated from the complex, resulting in nuclear translocation [21–24].

Here, we established novel inducible CRISPRa/i systems named iCRISPRa/i, constructed by fusing two ERT2 domains to the N terminal and one ERT2 domain to the C terminal of CRISPRa/i, respectively. iCRISPRa/i systems could effectively achieve exogenous and endogenous transcriptional regulation with 4OHT induction in multiple cell lines. Moreover, iCRISPRa/i systems have regulatory specificity at endogenous on-target sites. Besides, transcriptional regulation induced by 4OHT was dose-dependent and multiplex with tandem sgRNA array. We also demonstrated that drug responses of iCRISPRa/i systems were rapid and reversible. The efficiencies of gene expression regulation of iCRISPRa/i were comparable to those of non-inducible and doxycycline-inducible counterparts. Compared to TRE-CRISPRa/i systems, another drug-inducible CRISPRa/i system, iCRISPRa/i exhibit a lower leakage and a faster drug response activity. More importantly, iCRISPRa/i could be utilized to induce phenotypic changes in multiple mammalian cells, demonstrating its broader applicability, including but not limited to gain- and loss-of-function model construction and precise gene therapy.

## Materials and methods

### Plasmids construction

KRAB-dCas9-NLS-NLS and NLS-dCas9-VPR-NLS-NLS sequences were cloned from pHR-SFFV-KRAB-dCas9-P2A-mCherry [9] (gifted from Jonathan Weissman, Addgene plasmid # 60954) and SP-dCas9-VPR plasmid (obtained from MiaoLingBio, China, P1290), respectively. ERT2 sequence was cloned from the Nestin-Cre/ERT2 transgenic mice (The Jackson Laboratory Stock No: 016261) genomic DNA gifted from Yangling Mu. The CRISPRa/i-expressing plasmids, pcDNA3.1-CMV-CRISPRa/i-*myc*-His, were cloned by inserting KRAB-dCas9-NLS-NLS and NLS-dCas9-VPR-NLS-NLS into pcDNA3.1/*myc*-His A (Thermo Fisher Scientific Inc, V80020) under the control of CMV promoter. ERT2-CRISPRa/i variants were constructed by inserting KRAB-dCas9 or dCas9-VPR sequences with different ERT2 domains into the linearized pcDNA3.1/*myc*-His A vector. PB-TRE-dCas9-VPR plasmid (purchased from NovoPro, China, # V000906) and pHAGE TRE dCas9-KRAB plasmid (gifted from Rene Maehr and Scot Wolfe, Addgene plasmid # 50917) were used to express TRE-CRISPRa and TRE-CRISPRi constructs, respectively. The pcDNA3.1-2A-GFP reporter plasmid used in fluorescence imaging and flow cytometry to examine transcriptional regulatory activities was constructed by inserting the 2A-GFP sequence cloned from dCas9-VP64-GFP [25] (gifted from Feng Zhang, Addgene plasmid # 61422) to the downstream of CMV promoter of pcDNA3.1/*myc*-His A. pHR-CMV-iCRISPRa/i-2A-mCherry plasmids were constructed by removing SFFV promoter and KRAB-dCas9-NLS-NLS sequences from pHR-SFFV-KRAB-dCas9-P2A-mCherry and then inserting CMV promoter and iCRISPRa/i sequences through ClonExpress II One Step Cloning Kit (Vazyme, C112-02). The sgRNA candidates for transcriptional regulation listed in Table S1 were chosen from Genome-wide libraries for CRISPRi (Dolcetto) and CRISPRa (Calabrese) (https://wermelinglab.com/2018/10/05/genome-wide-libraries-for-crispri-dolcetto-and-crispra-calabrese/) or based on the prediction of high efficiency and low off-target effect by the computational analysis using CRISPOR [26] (http://crispor.gi.ucsc.edu/). They were inserted into a small gRNA scaffold of pU6-sgRNA (a gift from Yan Zhou) and pU6-sgRNA EF1Alpha-puro-T2A-BFP (gifted from Jonathan Weissman, Addgene plasmid # 60955) as described in the previous studies [27, 28]. One sgRNA displaying the best activity was chosen for transcriptional regulation. sgRNA array plasmids were constructed by cloning the sequences of U6 promoter and small guide RNAs and inserting them into linearized pU6-sgRNA vector using ClonExpress II One Step Cloning Kit. pUC-sgRNA-CRISPRa/i-2A-GFP plasmids and pUC-sgRNA-iCRISPRi-2A-GFP plasmid were constructed by cutting Cas9 from pX330-U6-Chimeric_BB-CBh-hSpCas9 [29] (gifted from Feng Zhang, Addgene plasmid # 42230) and replacing Cas9 with CRISPRa/i and iCRISPRi using ClonExpress II One Step Cloning Kit, and sgRNAs were inserted as described [27].

### Cell culture and transfection

HEK 293T cells (SCSP-502) were obtained from the National Collection of Authenticated Cell Cultures, NIH/3T3 cells were gifted from Yan Zhou, Wuhan University, Wuhan, China. B16 murine melanoma cells (CL-0029) were purchased from Wuhan Pricella Biotechnology Co., Ltd. C2C12 myoblasts were gifted by Zhenge Luo from ShanghaiTech University, Shanghai, China. HEK 293T cells and NIH/3T3 cells were cultured in high glucose Dulbecco’s modified Eagle’s medium (DMEM; Gibco), supplemented with 10% fetal bovine serum (FBS; ExCell Bio, FSP500) and 1% penicillin-streptomycin. B16 murine melanoma cells were cultured in Roswell Park Memorial Institute (RPMI) 1640 supplemented with 10% FBS and 1% penicillin-streptomycin (Pricella, CM-0029). When melanin synthesis was required, RPMI 1640 was replaced with DMEM containing L-tyrosine. C2C12 myoblasts were passaged and differentiated into myotubes as described previously [30]. Briefly, C2C12 cells were maintained as undifferentiated myoblasts in high glucose DMEM supplemented with 20% FBS and 1% penicillin-streptomycin. The fusion of myoblasts into myotubes was induced by culturing in the fusion medium: DMEM supplemented with 2% horse serum (Gibco, 26050088) and 1% penicillin-streptomycin. Before full differentiation, the fusion medium should be replaced every day. All cells were cultured in a humidified atmosphere with 5% CO_2_ at 37 °C. When necessary, cells were cultured in the media supplemented with either ethanol, 4OHT (Sigma-Aldrich, H6278), or doxycycline (MeilunBio, MB1088) at varying concentrations or for different durations of treatment.

Transfection for HEK 293T cells was performed using polyethylenimine (PEI, MW 25,000, Sigma-Aldrich, 408727) transfection reagent and transfection for B16 melanoma cells was performed using Zlip2000 transfection reagent (Zomanbio, ZC305) at around 80-90% cell confluence, according to the manufacturer’s instructions. Transfection for C2C12 myoblasts was performed using TransIntro^™^ PL Transfection Reagent (TransGen Biotech, FT301-01), as described previously [30]. Briefly, C2C12 myoblasts were seeded to 80-90% confluence and then incubated with a mixture of DNA, PL Transfection Reagent and Opti-MEM (Gibco, 31985070). The optimal dosage of the mixture for one 35 mm dish was 3 µg plasmid DNA and 9 µl PL dissolved in 800 µL Opti-MEM. After 6 h of incubation, the mixture was replaced by the fusion medium.

### Protein extraction and Western blot analysis

Cultured HEK 293T cells transfected with plasmids in 35 mm dishes were fractionated using the Rapid Efficient And Practical (REAP) method. Briefly, the cells were scraped in ice-cold PBS, collected into 1.5-mL microcentrifuge tubes, and centrifuged at 1,180× g for 5 min at 4 °C in a table-top centrifuge. After discarding the supernatant, the pellet was suspended with RIPA lysis buffer (Biosharp, BL504A) supplemented with 0.1 mM PMSF (Biosharp, BL507A) and treated with sonication for 2 cycles (each cycle consisting of 5 s of sonication followed by 5 s rest). Treating with ice-bath for 30 min, the sample was centrifuged at 13,780× g for 15 min at 4 °C and the supernatant was collected into a new tube as the “total protein”. For cell fractionation, the cell pellet was lysed with cytoplasmic lysis buffer containing 2% NP-40 (Biosharp, BS205) dissolved in PBS and supplemented with 0.1 mM PMSF. The lysate was suspended by pipette 5 times and then placed on ice for 5 min before being centrifuged at 1,000× g for 10 min at 4 °C. The resulting supernatant was collected into a new tube as the “cytoplasmic fraction”. Subsequently, the pellet underwent 3 cycles of sonication using nuclear lysis buffer [25 mM Tris-HCl (pH 7.4), 75 mM NaCl, 0.5 mM EDTA-Na_2_, 1% NP-40, 0.5% Triton X-100, 0.5% sodium deoxycholate and 0.05% SDS] supplemented with 0.1 mM PMSF. Finally, the sample was centrifuged at 21,000× g for 10 min at 4 °C and the supernatant was collected into a new tube as the “nuclear fraction”.

Samples were loaded and electrophoresed using SDS-PAGE and transferred to PVDF membranes. Subsequently, the membranes were incubated with the mouse anti-Myc antibodies (Proteintech China, 60003-2-Ig) for the detection of CRISPRa/i and iCRISPRa/i proteins, the rabbit anti-Cas9 antibodies (ABclonal Biotechnology, A14997) for the detection of doxycycline-inducible CRISPRa/i proteins, the mouse anti-α-tubulin antibodies (ABclonal Biotechnology, AC012) and the rabbit anti-LMNB1 antibodies (Proteintech China, 12987-1-AP). α-tubulin served as a cytoplasmic marker, and LMNB1 served as a nuclear marker. Finally, they were incubated with secondary antibodies and scanned by the ECL System (DNR). The band intensities of cytoplasmic and nuclear extracts were quantified using ImageJ software and normalized to α-tubulin (cytoplasmic marker) or LMNB1 (nuclear marker), respectively. The cytoplasmic-to-total and nuclear-to-total protein ratios were then determined by dividing the normalized intensity of each fraction by the sum of cytoplasmic and nuclear proteins.

### GFP expression assay

HEK 293T cells were cultured to reach 90% confluence in a 6-well culture plate and then co-transfected with GFP reporter plasmid, sgRNA plasmids and iCRISPRa/i plasmids simultaneously using PEI with a 20% proportion of GFP-positive cells per well. Twelve hours after transfection, the transfected cells were further cultured with fresh media supplemented with either ethanol or 1 µM 4OHT for 2 d. Subsequently, cells were washed with ice-cold PBS and fixed using 4% PFA for 30 min. Then fixed cells were stained with 2 µg/mL DAPI served as a control. The GFP and DAPI signals were observed under an inverted fluorescence microscope. The integrated density of GFP was rectified by DAPI signal and normalized based on the average integrated density of cells transfected with a plasmid expressing scrambled sgRNA without 4OHT treatment.

### GFP flow cytometry analysis

HEK 293T cells were co-transfected with GFP reporter plasmid, sgRNA plasmids and iCRISPRa/i plasmids as described above. After ethanol or 4OHT treatment for 2 d, cells were trypsinized, neutralized, collected by centrifuge for 2 min at 1,000× g, and the cell pellets were resuspended in 300 µL of PBS. Approximately 100,000 GFP-positive cells were counted by ID7000 Spectral Cell Analyzer (Sony Biotechnology, Japan) for each sample. The data were analyzed using FlowJo software (version 10.8.1, BD, USA). Three separate samples were recorded for each experiment. Graphs are representative data sets from one of three independent experiments.

### RNA extraction and quantitative PCR

Total RNA was extracted from HEK 293T cells, NIH/3T3 cells and B16 cells with TRIpure reagent (Keep Biotechnologies, RN0102) according to the manufacturer’s instructions. For each sample, total RNA was reverse transcribed by All-In-One 5× RT MasterMix with gDNA Removal (Applied Biological Materials Inc, G592). Transcriptional levels were quantified by qPCR using 2× Universal SYBR Green Fast qPCR Mix (ABclonal Biotechnology, RK21203) as manufacturer’s instructions. The qPCR reactions were run and analyzed in the Quantagene q225 instrument (Kubo Technology). Relative mRNA expression folds were determined by normalizing to the housekeeping gene *Gapdh*. The primer details are listed in Table S2.

### Lentivirus production and stable cell lines generation

Lentiviral preparations were produced in the HEK 293T cells using the 2nd generation psPAX2 and pCMV-VSVG packaging system in combination with either iCRISPRa/i or sgRNA transfer plasmids according to the manufacturer’s protocol. Cell culture media containing lentiviruses were first cleared by low-speed centrifugation and filtered through a 0.45 μm filter. Then, lentiviral particles were precipitated from the lentiviral preparation using 10% polyethylene glycol 8000 followed by centrifugation. The pellets containing viral particles were then resuspended in PBS, aliquoted, snap-frozen in liquid nitrogen and stored at -80 °C.

Before generating stable cell lines, cells were seeded and lentivirus infections were performed at around 90% cell confluence. Stable cell lines were generated through a two-step lentiviral transduction, first by iCRISPRa/i lentiviruses to establish the basal iCRISPRa/i systems, then by sgScr or sgRNA lentiviruses for specific gene targeting. Cells were cultured in the media containing 200 µL lentivirus suspension and polybrene with a final concentration of 8 µg/mL for 24 h. Subsequently, the media were replaced with fresh growth media containing 10 µg/mL puromycin for the selection of iCRISPRa/i stable cell lines or 1,000 µg/mL G418 for the selection of iCRISPRa/i-sgRNA stable cell lines. After 2 d of puromycin selection or 6-8 d G418 selection, regular medium changes were performed for cell growth. As polyclonal populations of resistant cells emerged and the individual cells became confluent, they were passaged into larger culture dishes.

### Determination of iCRISPRa/i activities at predicted Cas9 off-target sites

Off-target loci were predicted by Off-Spotter (https://cm.jefferson.edu/Off-Spotter/). Total RNA was extracted and reverse transcribed after 4OHT treatment as described above and the transcriptional alterations of off-target genes were quantified by qPCR using the primer sequences provided in Table S2. The off-target sites and their corresponding genes are listed in Table S3.

### RNA-Seq analysis

HEK 293T cells were co-transfected with iCRISPRa/i and sgRNA plasmids and treated with either ethanol or 4OHT as above description. After 2 d treatment of 4OHT, total RNA was extracted. The libraries were prepared as follows. Firstly, mRNA was purified with poly-T oligo-attached magnetic beads and then fragmented using divalent cations in First Strand Synthesis Reaction Buffer (5×). Secondly, using fragmented mRNA as a template, first strand cDNA was synthesized utilizing random hexamer primer and M-MuLV Reverse Transcriptase (RNase H-), and the Second strand cDNA synthesis was carried out using DNA Polymerase I and RNase H subsequently. Thirdly, the purified second-strand cDNA was processed and cDNA fragments with 370-420 bp in length were selected and amplificated through PCR. Finally, PCR products were purified using AMPure XP system and libraries were quantified using Agilent Bioanalyzer 2100 system. The libraries were sequenced on an Illumina NovaSeq X plus platform at the Novogene Bioinformatics Institute (Beijing, China). Data processing and bioinformatics analysis, including differential expression analyses, Venn diagrams analyses and GO enrichment analyses are performed by use of the NovoMagic platform. Differential expression analyses of target genes were performed using the DESeq2 R package (1.20.0). Significantly differentially expressed genes were defined as genes whose expression level|log2(fold change)| > 1 and *p* < 0.05 in the RNA-Seq of *Klf4* transcripts and genes whose expression level|log2(fold change)| > 0.34 and *p* < 0.05 in the RNA-Seq of *Prdm4* transcripts. A *p*-value < 0.05 was considered a significant difference in Venn diagrams and GO enrichment analyses.

### Tyrosinase activity and melanin content assays

The RPMI 1640 medium was replaced with DMEM to facilitate melanin synthesis in B16 cells, and the cells were subsequently cultured for an additional 2 d. B16 cells were lysed using RIPA lysis buffer supplemented with 0.1 mM PMSF for 30 min at 4 ℃, and then subjected to 2 cycles of sonication (each cycle consisted of 5 s of sonication followed by 5 s of rest), and then the lysate was centrifuged at 21,000× g for 10 min at 4 °C. The supernatant containing total protein was quantified by measuring optical density at 560 nm using the BCA method. Then 20 µL of supernatant containing 10 µg total protein was mixed with 100 µL of 0.1% L-DOPA (Sigma-Aldrich, D9628) in PBS (pH 6.8) and added to each well in a 96-well plate. After incubation at 37 °C for 1 h, the tyrosinase activity was monitored by measuring the absorbance at 490 nm. The pellet containing melanin was dissolved in 1 M NaOH/10% DMSO solution for 1 h at 90 ℃. Melanin content was measured as optical density at 490 nm using a microplate reader (Tecan) and normalized to total protein content.

### C2C12 myoblast fusion assay

The C2C12 myoblasts were cultured until reaching 90% confluence in the growth medium. Subsequently, the myoblasts were transfected. After 6 h of transfection, the cells were incubated in the fusion media containing either ethanol or 1 µM 4OHT on day 1 and day 2. Following an additional two-day period, the cells were fixed using a solution of 4% PFA. Then, they were permeabilized with a solution of 0.03% Triton X-100 and blocked with a solution of 5% BSA in PBS for 2 h. The cells were then subjected to overnight incubation with rabbit polyclonal antibodies against Desmin (diluted at a ratio of 1:100; ABclonal Biotechnology, A3736) in a solution containing 5% BSA. Afterward, the cells underwent three washes with ice-cold PBS and subsequent incubation with anti-rabbit secondary antibodies conjugated with Rhodamine (TRITC) (diluted at a ratio of 1:200; Proteintech China, SA00007-2). Nucleus staining was performed using DAPI at a concentration of 2 µg/mL dissolved in PBS for 10 min. Finally, visualization was carried out using Zeiss fluorescence microscope equipped with ZEN imaging system software. ImageJ software provided by National Institutes of Health (Bethesda, MD) was employed for analysis purposes to quantify various parameters related to myogenic differentiation including fusion index, maturation index, the area of total GFP-positive cells, length and width measurements of individual GFP-positive cells along with the enumeration of cells containing different ranges of nuclei (1–3 nuclei, 4–7 nuclei and 8 + nuclei). The fusion index was acquired by calculating the percentage of nuclei within the GFP-positive cells containing more than three nuclei to the number of nuclei of total GFP-positive cells. The maturation index was calculated as the percentage of GFP-positive cells containing more than five nuclei to the total number of GFP-positive cells.

### Statistical analysis

Each experiment was repeated in three biological replicates. All the quantitative data were presented as the mean ± standard deviation (SD). All statistical analyses were carried out using GraphPad Prism 8.0 (GraphPad Software, San Diego, CA, USA). Data were analyzed using an unpaired *t*-test (for comparisons with one independent variable and two groups), a one-way analysis of variance (ANOVA) (for comparisons with one independent variable and > 2 groups), or a two-way ANOVA (for comparisons with two independent variables and > 2 groups). A *p* < 0.05 was considered statistically significant.

## Results

### Development of optimal drug-inducible CRISPRa/i variants

**Fig. 1:**
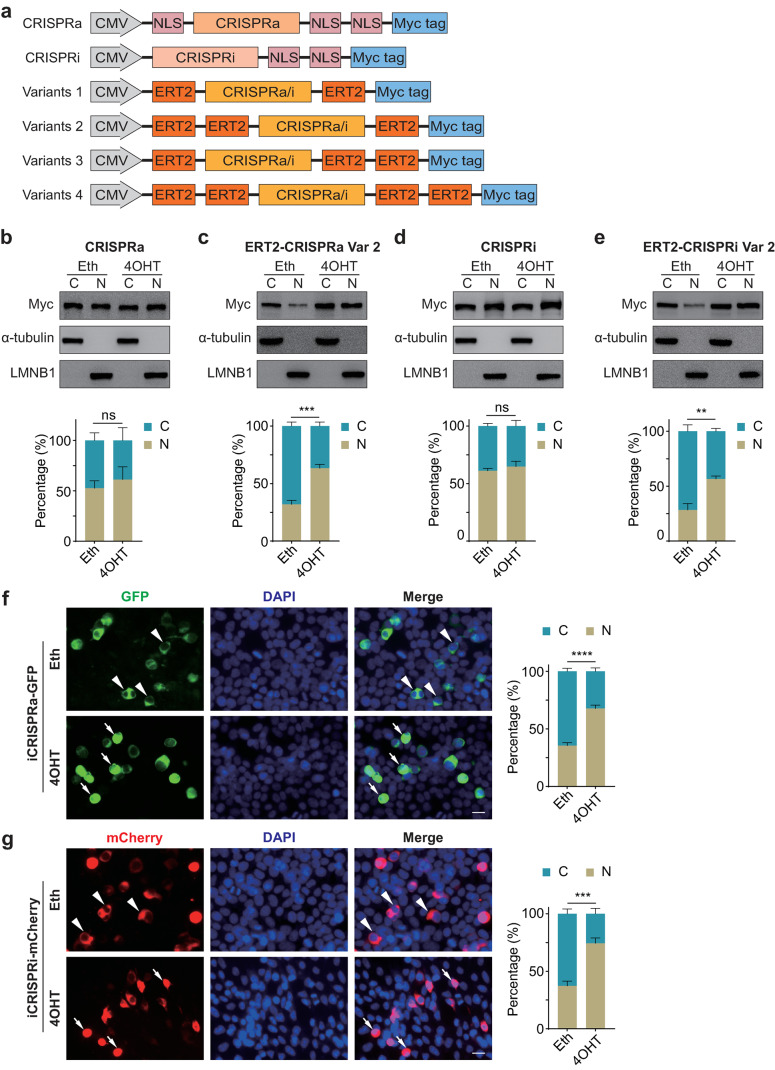
Development of optimal drug-inducible CRISPRa/i variants. **a** Architectures of CRISPRa/i and different ERT2-CRISPRa/i variants. The top two rows are the referenced CRISPRa and CRISPRi constructs, which contain Myc epitope tags and NLS domains joined to dCas9-effectors. Other rows represent four distinct configurations of ERT2 domain and dCas9-effectors that were evaluated in this study. **b-e** Western blot and quantification of subcellular localization of CRISPRa/i **b**,** d** and ERT2-CRISPRa/i variants 2 **c**,** e**. Transfected HEK 293T cells were separated into cytoplasmic and nuclear fractions after ethanol or 4OHT treatment for 24 h. CRISPRa/i and ERT2-CRISPRa/i variants 2 tagged with Myc tags could be detected with anti-Myc antibodies. α-tubulin served as a cytoplasmic marker, while LMNB1 served as a nuclear marker. Var: variant. Eth: ethanol. C: cytoplasmic fraction, N: nuclear fraction. **f**,** g** Fluorescence images and quantification of the percentage of cells containing cytoplasmic- and nuclear-located iCRISPRa **f** and iCRISPRi **g** proteins. iCRISPRa protein was tagged with GFP reporter and iCRISPRi protein was tagged with mCherry reporter to monitor their subcellular localization. Transfected HEK 293T cells were treated with ethanol or 4OHT for 24 h, and the nuclei were stained with DAPI. Eth: ethanol. Arrowheads indicate that iCRISPRa/i proteins were localized at the cytoplasm, and arrows indicate that iCRISPRa/i proteins were localized at the nucleus. At least 600 cells were counted for each group. Scale bars, 20 μm. All photographs shown in this figure are representative of three independent experiments. Data are presented as mean ± SD, *n* = 3 biological replicates. ns, not significant (*p* > 0.05), ** *p* < 0.01, *** *p* < 0.001, **** *p* < 0.0001; Student’s *t*-test

Cre recombinase fused with an ERT2 domain has been widely used for inducible gene ablation with 4OHT treatment [31–34], therefore we hypothesized that fusing ERT2 domains to CRISPRa/i might render the nuclear translocation of the fusion proteins dependent on 4OHT. We started by constructing four different variants of CRISPRa/i fused with varying amounts of ERT2 domain (ERT2-CRISPRa/i). Since more ERT2 domains are required for larger proteins to achieve tighter regulation [18], we removed the nuclear localization signal (NLS) domains and inserted one or two ERT2 domains instead at either the N or C terminus of CRISPRa/i proteins tagged with Myc tag to gain an inducible transcriptional regulatory effect (Fig. 1a). To evaluate whether the ERT2-CRISPRa/i fusion proteins could respond to 4OHT induction, we performed subcellular location analysis by Western blot to determine the changes of the cytoplasmic and nuclear distribution of our designed ERT2-CRISPRa/i variants in the presence or absence of 4OHT. These constructs were transfected into HEK 293T cells for 24 h of overexpression followed by additional ethanol or 1 µM 4OHT treatment for 24 h. Cytoplasmic and nuclear fractions of transfected HEK 293T cells were separated using the REAP protocol [35]. The data showed no significant differences in the cytoplasmic and nuclear distributions of CRISPRa protein after either ethanol or 4OHT treatment (Fig. 1b), however, the cytoplasmic and nuclear distributions of ERT2-CRISPRa variants differed variously. For variant 1, the percentage of nuclear-localized fusion protein accounted for 23.82% of the total protein before the 4OHT treatment; with the 4OHT treatment, this percentage changed to 31.43% (Fig. S1a). These data showed that the redistribution efficiency of ERT2-CRISPRa-ERT2 was limited upon exposure to 4OHT. Both variants 2 and 3 contain three ERT2 domains, but the distinct arrangement of the ERT2 domains at the N and C terminus may affect the spatial structure of fusion proteins, resulting in different protein subcellular distributions. Without 4OHT treatment, fusion proteins from both variants exhibited limited segregation in the nucleus due to the retention of ERT2 domains, with approximately 30% nuclear localization. Upon 4OHT stimulation, variant 2, ERT2-ERT2-CRISPRa-ERT2, exhibited a significant increase in nuclear localization, reaching 63.26% (Fig. 1c), resembling that of non-inducible CRISPRa; whereas variant 3 (ERT2-CRISPRa-ERT2-ERT2) showed no significant nuclear relocation (Fig. S1b). For variant 4, ERT2-ERT2-CRISPRa-ERT2-ERT2, the fusion protein was further enriched in the cytoplasm without 4OHT treatment, potentially attributed to the further increased number of ERT2 domains. Upon 4OHT induction, no significant difference in the proportion of nuclear-localized fusion protein was observed (Fig. S1c). Meanwhile, the analyses of CRISPRi and ERT2-CRISPRi variants revealed that, similar to non-inducible CRISPRi whose protein subcellular distributions showed no significant difference before and after 4OHT induction (Fig. 1d), variant 2, ERT2-ERT2-CRISPRi-ERT2, exhibited the highest proportion of nuclear-localized protein with 4OHT treatment (Fig. 1e). Other variants showed no significant differences in the cytoplasmic and nuclear distributions upon either ethanol or 4OHT treatment (Fig. S1d-f). In summary, we verified that variants 2, ERT2-ERT2-CRISPRa/i-ERT2 fusions (named iCRISPRa/i), exhibited the most remarkable protein translocation from cytoplasm to the nucleus with 4OHT treatment, thus establishing their utility as drug-inducible CRISPRa/i systems for transcriptional regulation.

To verify the above results, we further examined the intracellular localization of iCRISPRa/i proteins fused GFP or mCherry reporters using fluorescence microscopy following 4OHT induction. In the absence of 4OHT, a majority of iCRISPRa/i proteins were localized in the cytoplasm, with only 35.56% and 37.26% of cells exhibiting nuclear-localized iCRISPRa and iCRISPRi proteins, respectively. However, after 48 h of 4OHT treatment, there was a significant increase in the proportion of cells exhibiting nuclear-localized iCRISPRa and iCRISPRi protein to 67.74% and 74.26%, respectively (Fig. 1f and g). These findings demonstrate that the fusion of two ERT2 domains at the N-terminus and one ERT2 domain at the C-terminus of CRISPRa/i allows precise control of the intracellular localization of iCRISPRa/i proteins by sequestrating them in the cytoplasm in the absence of 4OHT and translocating them into the nucleus upon the addition of 4OHT.

Next, we used the doxycycline (Dox)-inducible CRISPRa/i (TRE-CRISPRa/i) systems to benchmark our new inducible systems. Similar to iCRISPRa/i plasmids, we transfected TRE-CRISPRa/i plasmids into HEK 293T cells and treated them with or without 1 µg/mL doxycycline for 24 h. Then, the expression of CRISPRa/i proteins was detected by Western blot and quantified. After doxycycline induction, the expression of CRISPRa/i increased significantly up to 11.87-fold and 8.52-fold. However, low levels of CRISPRa/i expression in the absence of doxycycline treatment were detected (Fig. S2a and b), suggesting protein expression leakage of Dox-inducible CRISPRa/i systems.

### Exogenous and endogenous genes could be regulated transcriptionally by iCRISPRa/i systems

**Fig. 2:**
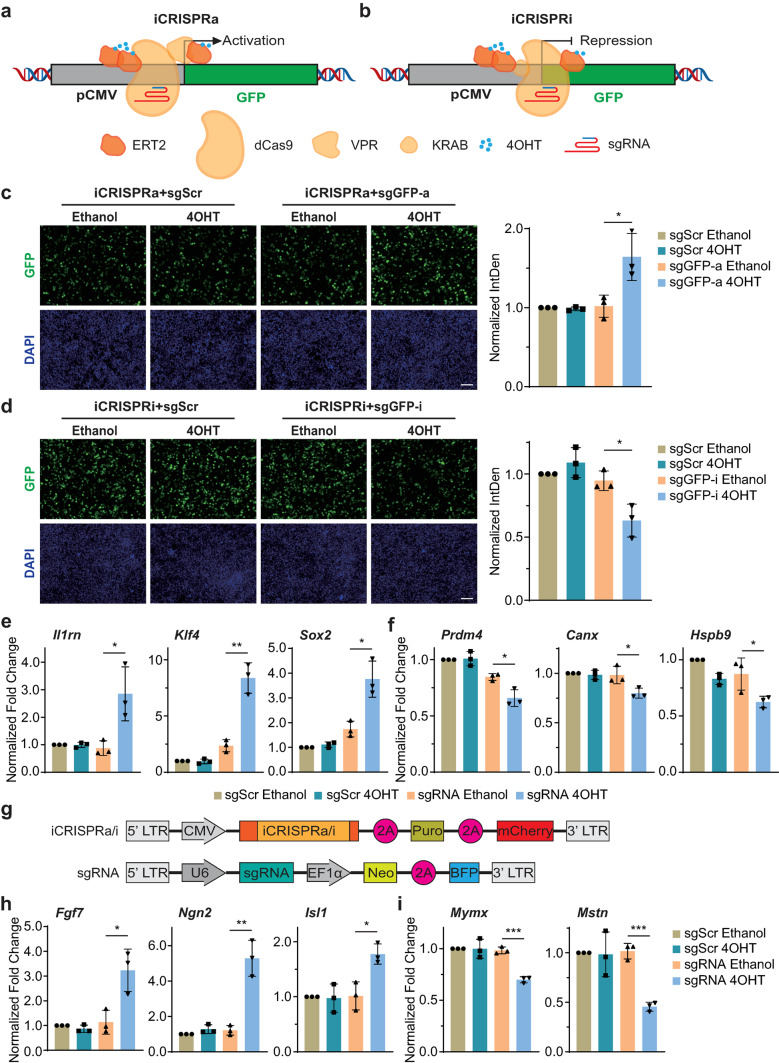
Exogenous and endogenous genes could be regulated transcriptionally by iCRISPRa/i. **a**,** b** Overview of exogenous GFP transcriptional activation **a** and repression **b** assays. HEK 293T cells were co-transfected with GFP reporter driven by CMV promoter, sgGFP-a/i targeting CMV promoter region of GFP and iCRISPRa/i plasmids and treated with either ethanol or 4OHT for 48 h. **c**,** d** Fluorescence images and quantification of the GFP signals were analyzed by the integrated density plugin in ImageJ of the cells transfected with iCRISPRa/i and appropriate sgGFP-a/i plasmids and normalized to DAPI. Data are shown as integrated density normalized to control samples. IntDen: integrated density. Scale bars, 100 μm. All photographs shown in this figure are representative of three independent experiments. Each data point represents an average of multiple fields of one experiment sample. **e**,** f** Detection of transcriptional regulation of endogenous genes by iCRISPRa **e** and iCRISPRi **f** with 4OHT induction in HEK 293T cells. *Il1rn*, *Klf4* and *Sox2* transcripts were targeted with the iCRISPRa system to activate transcription, *Prdm4*, *Canx* and *Hspb9* transcripts were targeted with the iCRISPRi system to repress transcription. **g** Schematic of lentivirus vectors encoding iCRISPRa/i and sgRNA used for generating stable cell lines with different resistance and fluorescence genes. **h**,** i** Detection of transcriptional regulation of endogenous genes by iCRISPRa **h** and iCRISPRi **i** with 4OHT induction in NIH/3T3 cells. *Fgf7*, *Ngn2* and *Isl1* transcriptional levels were activated by the iCRISPRa system, *Mymx* and *Mstn* transcriptional levels were repressed by the iCRISPRi system. Transcriptional levels of target genes of each sample were detected by qPCR and normalized to the housekeeping gene *Gapdh*. Data are presented as mean ± SD, *n* = 3 biological replicates. * *p* < 0.05, ** *p* < 0.01, *** *p* < 0.001; Student’s *t*-test

To determine whether the iCRISPRa/i could regulate exogenous gene transcription with 4OHT induction, we constructed a CMV-driven GFP reporter plasmid (pcDNA3.1-2A-GFP). HEK 293T cells were co-transfected with GFP reporter, sgRNAs against the CMV promoter region of GFP (sgGFP-a/i) and iCRISPRa/i plasmids (Fig. 2a and b). As shown in Fig. S3a and b, with the guidance of sgGFPa/i, the expression of GFP was up/downregulated by CRISPRa/i systems, suggesting the effectiveness of sgGFP-a/i. For the functional verification of iCRISPRa/i systems, after induction with 4OHT, transfected cells were fixed, and the GFP signal was observed using an inverted fluorescence microscope and analyzed by the integrated density plugin in ImageJ. The iCRISPRa/i systems showed effective transcriptional regulatory activity on CMV-driven GFP expression in HEK 293T cells when targeted to the CMV promoter region under the guidance of sgGFP-a/i. Compared with the ethanol-treated groups, upon 4OHT induction, cells expressing iCRISPRa/i and sgGFP-a/i exhibited a significant 1.61-fold increase and a modest 33.34% decrease in GFP expression, respectively (Fig. 2c and d). These results indicate that exogenous gene transcription was effectively regulated by iCRISPRa/i under 4OHT induction. However, compared with non-inducible CRISPRa/i systems, the transcriptional regulation efficiencies of iCRISPRa/i were lower. This may be due to the lower expression efficiencies of iCRISPRa/i, which have larger sizes. The regulation efficiencies of iCRISPRa/i systems upon GFP up/down regulation were further analyzed by GFP flow cytometry analysis. As shown in Fig. S4a and b, we found that iCRISPRa/i systems achieved a 1.44-fold increase in GFP expression activation and a 30.04% decrease in GFP expression repression, which were similar to the transcriptional regulation efficiencies shown in Fig. 2c and d.

Next, to evaluate the ability of iCRISPRa/i to modulate endogenous gene transcription, we assessed the transcriptional levels of endogenous genes altered by iCRISPRa/i upon 4OHT treatment in HEK 293T cells. *Il1rn*, *Klf4* and *Sox2* were chosen for activation verification, and *Prdm4*, *Canx* and *Hspb9* for repression verification. Our results showed that the CRISPRa/i systems could regulate the transcription of target genes mentioned above under the guidance of target sgRNAs (Fig. S5a and b). Then HEK 293T cells were co-transfected with iCRISPRa/i and sgRNA plasmids and treated with either ethanol or 4OHT for 48 h. The quantitative PCR (qPCR) detection of target gene transcripts revealed varying degrees of transcriptional regulation in the presence of 4OHT. Overall, iCRISPRa system showed significant activation activities at different target sites. Compared with ethanol treatment, 4OHT induction generated significant transcriptional activation of target genes in the cells transfected with iCRISPRa and target sgRNA plasmids, there was a 3.51-fold increase in *Il1rn* expression, a 3.68-fold increase in *Klf4* expression and a 2.34-fold increase in *Sox2* expression (Fig. 2e). Moreover, 4OHT induction also exhibited transcriptional repression of target genes in the cells expressing iCRISPRi and target sgRNAs, resulting in a 22.2% decrease in *Prdm4* expression, an 18.1% decrease in *Canx* expression and a 28.2% decrease in *Hspb9* expression (Fig. 2f). The regulatory activities were comparatively lower than those reported for non-inducible CRISPRa/i in previous studies, we attribute these discrepancies to the lower percentage of transfected cells [4, 36] and the guidance of single sgRNA [37]. However, there was no significant difference between ethanol-treated and 4OHT-treated cells transfected with iCRISPRa/i and scrambled sgRNA (sgScr) plasmids. These data suggested that iCRISPRa/i systems could effectively regulate the transcription of target genes in HEK 293T cells. The transcriptional regulation efficiencies of iCRISPRa/i were comparable to non-inducible and Dox-inducible CRISPRa/i systems (Fig. S6a and b). Compared with CRISPRa/i systems, iCRISPRa/i and TRE-CRISPRa/i systems exhibited comparable efficiencies in the modulation of endogenous genes, *Klf4* and *Prdm4*, upon treatment with inducers. However, we observed no significant difference in transcriptional regulation between non-treated and Dox-treated cells expressing target sgRNAs in TRE-CRISPRa/i systems, suggesting the marked leakage of TRE-CRISPRa/i systems in transiently transfected cells, which was not reported in stable cell lines established by lentivirus infection or transposition [9, 38]. These data showed that compared with TRE-iCRISPRa/i systems, iCRISPRa/i systems could have broader applications in transcriptional regulation.

We further assessed the applicability of iCRISPRa/i systems in NIH/3T3, a mouse fibroblast cell line widely used in molecular biology studies. We established stable NIH/3T3 cell lines expressing iCRISPRa/i and sgRNA by lentivirus infection to overcome the delivery challenges associated with the large sizes of iCRISPRa/i plasmids. iCRISPRa/i lentiviruses contain CMV promoter-driven iCRISPRa/i fused with puromycin resistance gene (Puro) and mCherry in tandem via two self-cleaving 2A peptides [39]. Similarly, the sgRNA lentiviruses were driven by mouse U6 promoter and co-expressed with EF1α promoter-driven neomycin resistance gene (Neo) and BFP in tandem via a 2A peptide (Fig. 2g). After lentivirus infection, the stable cell lines were established through antibiotic selection. First, we analyzed the subcellular distribution of iCRISPRa/i proteins in stable NIH/3T3-iCRISPRa/i cells. With ethanol treatment, most iCRISPRa/i proteins were localized in the cytoplasm. Upon 4OHT induction, the proportion of nucleus-localized iCRISPRa/i proteins increased significantly (Fig. S7a and b), suggesting that iCRISPRa/i proteins could translocate from the cytoplasm to the nucleus in stable NIH/3T3-iCRISPRa/i cells, consistent with observations in transiently transfected HEK 293T cells. Second, to avoid cell line variability due to the differential expression of sgRNAs, we examined the transcriptional levels of control and target sgRNAs in NIH/3T3-iCRISPRa/i-sgRNA cells. As shown in Fig. S7c and d, sgRNA expression levels showed no difference between sgScr and target sgRNAs under either ethanol or 4OHT treatment. Then, we examined whether iCRISPRa/i could regulate the endogenous gene transcription in these cells. *Fgf7*, *Ngn2* and *Isl1* were selected for activation verification, while *Mymx* and *Mstn* were chosen for repression verification. The NIH/3T3-iCRISPRa/i cell lines infected with target sgRNA lentiviruses generated significant transcriptional regulatory effects upon 4OHT induction, compared to those treated with ethanol, whereas the sgScr-infected NIH/3T3-iCRISPRa/i cell lines displayed no significant change in response to either ethanol or 4OHT treatment. Regarding transcriptional activation, iCRISPRa effectively activates the transcriptional levels of different target genes upon 4OHT induction, showing an increase of 2.99-fold in *Fgf7* expression, an increase of 4.70-fold in *Ngn2* expression and an increase of 1.81-fold in *Isl1* expression (Fig. 2h); for transcriptional repression, iCRISPRi successfully suppressed the transcriptional levels of target genes in the presence of 4OHT, resulting in a decrease of 28.94% in *Mymx* expression and a decrease of 55.27% in *Mstn* expression (Fig. 2i). The observed transcriptional regulatory activities of iCRISPRa/i were different from that in plasmid transiently transfected cells, which may be due to the lower expression efficiency in lentivirus-infected cells [18]. These data suggested that iCRISPRa/i can effectively regulate the transcription of target genes through plasmid transient transfection and lentivirus infection under 4OHT induction in human and mouse cells.

### Targeting specificity of iCRISPRa/i

**Fig. 3:**
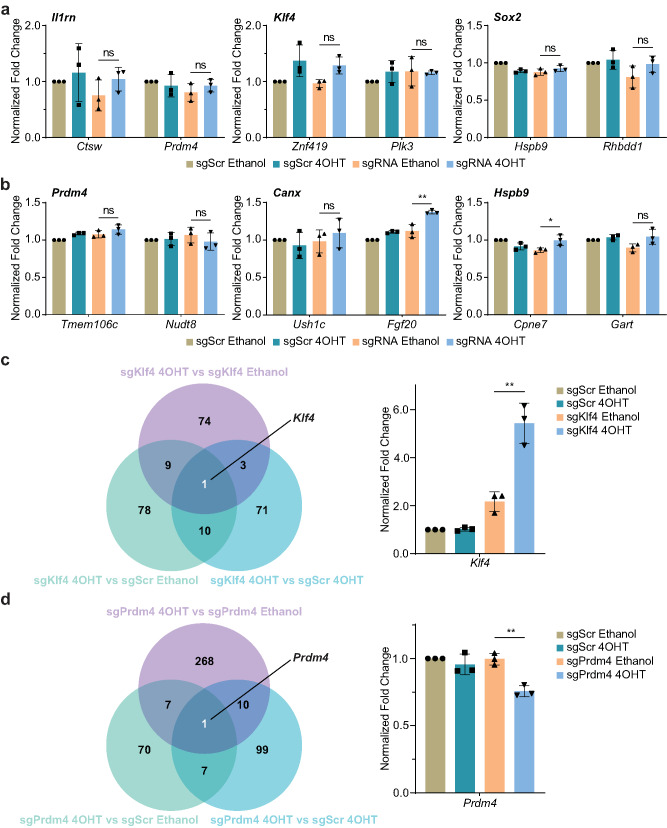
Targeting specificity of iCRISPRa/i. **a**,** b** The transcriptional levels of predicted iCRISPRa **a** and iCRISPRi **b** off-target genes were measured in transfected HEK 293T cells with ethanol or 4OHT treatment. On-target genes were labeled above the figures. Transcriptional levels of target genes of each sample were detected by qPCR and normalized to the housekeeping gene *Gapdh*. **c**,** d** Venn diagrams showed the overlap of regulated genes among different groups achieved by iCRISPRa **c** and iCRISPRi **d**, with common regulated genes listed, and the normalized fold changes of regulated genes were quantified. Different colored circles represent different comparison combinations. The overlapping regions in the two different-colored circles represent the number of different genes shared by the two comparison combinations. Data are presented as mean ± SD, *n* = 3 biological replicates. ns, not significant (*p* > 0.05), * *p* < 0.05, ** *p* < 0.01; Student’s *t*-test

To gain insights into the specificity of the iCRISPRa/i systems, we first measured the transcriptional levels of the predicted off-target genes. Off-Spotter (https://cm.jefferson.edu/Off-Spotter/) was applied to predict potential off-target sites [40]. Based on the most effective sgRNA window reported in the previous study [9], two potential off-target genes were selected for each sgRNA which contained the predicted off-target sites near their promoter regions and had the highest scores in the prediction. Subsequently, transfected HEK 293T cells were treated with either ethanol or 4OHT, and the transcriptional levels of the two potential off-target genes for each target sgRNA were detected by qPCR. Compared with the specific transcriptional activation and repression of the on-target gene of each sgRNA upon 4OHT induction shown in Fig. 2e and f, the transcription of the potential off-target genes showed no significant transcriptional activation or repression between groups with or without 4OHT treatment in cells transfected with target sgRNAs (Fig. 3a and b), suggesting there was no transcriptional regulation activities by iCRISPRa/i at predicted off-target sites.

We further performed genome-wide RNA sequencing (RNA-Seq) analysis to evaluate the specificity of iCRISPRa/i targeting. HEK 293T cells were co-transfected with iCRISPRa/i and sgRNA plasmids, and total RNA was extracted for RNA-Seq following either ethanol or 4OHT treatment. In the iCRISPRa system, upon 4OHT induction, 87 genes exhibited transcriptional alteration of at least two-fold compared to ethanol treatment in sgKlf4-transfected cells, 85 genes displayed transcriptional alteration relative to sgScr-transfected cells treated with 4OHT, and 98 genes showed transcriptional alteration relative to sgScr-transfected cells treated with ethanol. These differentially expressed genes (DEGs) are listed in Supplementary Data 2. To exclude the potential confounding gene transcriptional alteration induced by 4OHT, the metabolite of tamoxifen which is the analogue of widely distributed estrogen, we examined the intersection of these three comparisons and found only one overlapping gene, *Klf4*, which had 2.51-fold activation after 4OHT treatment in sgKlf4-transfected cells relative to that with ethanol treatment (Fig. 3c). In the iCRISPRi system, upon 4OHT induction, 286 genes exhibited transcriptional alterations compared to ethanol treatment in sgPrdm4-transfected cells, 117 genes displayed transcriptional alteration relative to sgScr-transfected cells with 4OHT treatment, and 85 genes showed transcriptional alteration relative to sgScr-transfected cells treated with ethanol. These DEGs were listed in Supplementary Data 3. Similar to iCRISPRa system, there was only one overlapping gene in the intersection of these three comparisons, *Prdm4*, which had a 24.06% decrease after 4OHT treatment in sgPrdm4-transfected cells relative to that with ethanol treatment (Fig. 3d).

To illustrate the character of iCRISPRa/i systems, we also analyze the DEGs found in RNA-Seq. Estrogen is a steroid compound and plays crucial roles in regulating the cardiovascular system, liver, pancreas, bone, brain, immune system, and spermatogenesis [41]. Estrogen-mediated signaling is involved in various physiological processes, such as regulation of cell differentiation, proliferation, and apoptosis, changes in gene expression, cAMP regulation, and protein-kinase activation of signaling cascades [42]. As an estrogen analogue, tamoxifen competitively binds to ERs and reduces estrogen-induced effects [43]. Thus, we hypothesized that the DEGs might be associated with the 4OHT treatment. We performed Gene Ontology (GO) enrichment analysis to classify these DEGs into different functional categories. The top 10 significantly enriched terms among DEGs identified in iCRISPRa/i systems within biological process (BP) categories were presented in Fig. S8 and Fig. S9 and listed in Supplementary Data 4 and 5. The vast majority of BP were functionally related to 4OHT exposure, such as natural killer cell activation and differentiation (GO:0032816, GO:0032823), spindle assembly (GO:0051225, GO:0007051, GO:0000212), osteoblastogenesis and mineralization (GO:0030282, GO:0031214, GO:0035265, GO:0045778), carbohydrate transport (GO:1904659, GO:0008645, GO:0034219, GO:0015749, GO:0008643), response to estrogen (GO:0043627), in utero embryonic development (GO:0001701), ATP synthesis (GO:0006120, GO:0042775, GO:0042773, GO:0022904, GO:0006119, GO:0022900, GO:0045333, GO:0010257, GO:0032981), negative regulation of neuron death (GO:1901215, GO:0043524), MAPK activity (GO:0043406, GO:0000187), epithelial to mesenchymal transition (GO:0010717, GO:0010718) and more, suggesting these gene dysregulations likely stem from 4OHT treatment. The enriched BP terms also included immune response associated with T cells (GO:0002710, GO:0002827), which are likely related to inflammatory responses triggered by transient plasmid transfection, as reported previously [44]. Together, our findings demonstrate the high specificity in regulating gene expression achieved by iCRISPRa/i systems.

###  Transcriptional regulation induced by 4OHT was dose-dependent and multiplex

**Fig. 4:**
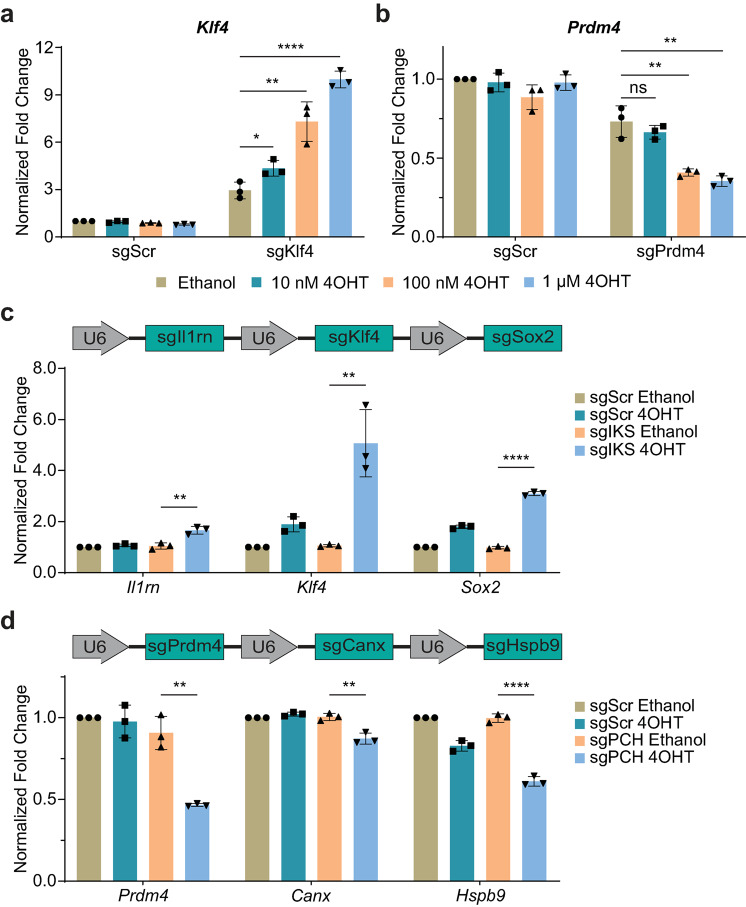
Transcriptional regulation induced by 4OHT was dose-dependent and multiplex. **a**,** b** Detection of the activation effect on *Klf4* transcript **a** and repression effect on *Prdm4* transcript **b** with different 4OHT dosages. Transfected HEK 293T cells were treated with ethanol, 10 nM, 100 nM, or 1 µM 4OHT and harvested at 48 h after treatment for qPCR. **c** Schematic of the sgRNA array targeting *Il1rn*, *Klf4* and *Sox2* genes and simultaneous activation of all targets by iCRISPRa with 4OHT inducement in transfected HEK 293T cells. **d** Schematic of the sgRNA array targeting *Prdm4*, *Canx* and *Hspb9* genes and simultaneous repression of all targets by iCRISPRi with 4OHT inducement in transfected HEK 293T cells. Transcriptional levels of target genes of each sample were detected by qPCR and normalized to the housekeeping gene *Gapdh*. Data are presented as mean ± SD, *n* = 3 biological replicates. ns, not significant (*p* > 0.05), * *p* < 0.05, ** *p* < 0.01, **** *p* < 0.0001; Student’s *t*-test

To further characterize the transcriptional regulation of endogenous genes induced by 4OHT, HEK 293T cells were transfected with iCRISPRa/i and sgRNA plasmids, followed by culturing in the media containing either ethanol or varying concentrations of 4OHT (10 nM, 100 nM and 1 µM). The transcriptional levels of target genes were detected by qPCR. The transcriptional levels of *Klf4* and *Prdm4* showed dose-dependent increases and decreases, with higher 4OHT concentrations yielding more significant changes in transcriptional levels. Contrasting with cells transfected with target sgRNAs, those transfected with sgScr showed no significant difference when exposed to different concentrations of 4OHT (Fig. 4a and b). Our data demonstrate that the transcriptional regulatory activities mediated by iCRISPRa/i were both dose-dependent and specifically induced by 4OHT treatment.

Given that gene networks regulate numerous physiological processes, achieving specific phenotypic changes often requires simultaneous regulation of multiple genes. The potent effectiveness of iCRISPRa/i inspired us to extend the application of the iCRISPRa/i systems in a multiplexed targeting manner. To characterize the feasibility of concurrent manipulation of multiple genes using iCRISPRa/i, we constructed sgRNA arrays targeting three distinct genes and examined the transcriptional levels of target genes in transfected cells by qPCR. Using sgRNA arrays, iCRISPRa/i simultaneously induced multiple transcriptional regulatory alterations upon 4OHT treatment. For iCRISPRa system, after 4OHT induction, target genes were simultaneously activated with a 1.61-fold increase in *Il1rn* expression, a 4.79-fold increase in *Klf4* expression and a 3.10-fold increase in *Sox2* expression using the sgIl1rn-sgKlf4-sgSox2 (sgIKS) array (Fig. 4c); for iCRISPRi system, expression of target genes was simultaneously repressed with a 49.14% reduction in *Prdm4* expression, an 11.60% reduction in *Canx* expression and a 38.80% reduction in *Hspb9* expression using the sgPrdm4-sgCanx-sgHspb9 (sgPCH) array upon 4OHT induction (Fig. 4d). More importantly, the sgRNA arrays showed comparable regulatory efficiencies on their respective targets compared to the individual sgRNA (Fig. 2e and f). These results highlighted the advantages and potential of using iCRISPRa/i systems for modulating complex genetic networks.

### Transcriptional regulation kinetics of iCRISPRa/i systems

**Fig. 5:**
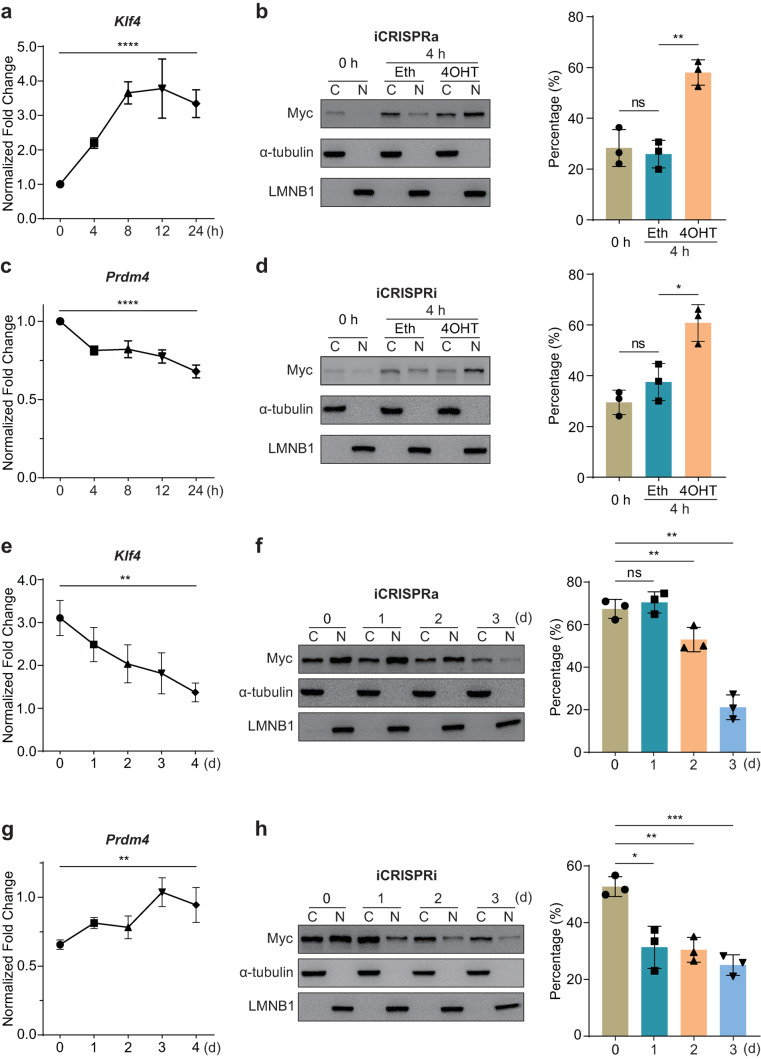
Transcriptional regulation kinetics of iCRISPRa/i systems. **a**,** c** Detection of *Klf4***a** and *Prdm4***c** transcriptional levels after 4OHT treatment with various durations. Transfected cells were treated with ethanol or 4OHT for 0 h, 4 h, 8 h, 12 h and 24 h, and harvested for qPCR. The ethanol-treated cells were regarded as the reference for 4OHT-treated cells at each time point. Transcriptional levels of target genes of each sample were detected by qPCR and normalized to the housekeeping gene *Gapdh*. Data are presented as mean ± SD, *n* = 3 biological replicates. **** *p* < 0.0001, one-way ANOVA. **b**,** d** Subcellular localization analyses of iCRISPRa **b** and iCRISPRi **d** with treatment of ethanol or 4OHT for 0 h and 4 h by Western blot. iCRISPRa/i proteins tagged with Myc tag could be detected with anti-Myc antibodies. α-tubulin served as a cytoplasmic marker, while LMNB1 served as a nuclear marker. Eth: ethanol. C: cytoplasmic fraction, N: nuclear fraction. Data are presented as mean ± SD, *n* = 3 biological replicates. ns, not significant (*p* > 0.05), * *p* < 0.05, ** *p* < 0.01; Student’s *t*-test. **e**,** g** Detection of *Klf4***e** and *Prdm4***g** transcriptional levels after 4OHT retraction. Transfected cells were treated with either ethanol or 4OHT for 2 d, then 4OHT was replaced by ethanol, and the transcriptional levels were detected by qPCR at 0 d, 1 d, 2 d, 3 d and 4 d. The ethanol-treated cells were regarded as the reference for 4OHT-treated cells at each time point. Transcriptional levels of target genes of each sample were detected by qPCR and normalized to the housekeeping gene *Gapdh*. Data are presented as mean ± SD, *n* = 3 biological replicates. ** *p* < 0.01, one-way ANOVA. **f**,** h** Subcellular localization analyses of iCRISPRa **f** and iCRISPRi **h** after 4OHT retraction. Transfected HEK 293T cells were treated with 4OHT for 24 h. After induction, 4OHT was replaced by ethanol and the subcellular localization was analysed by Western blot at 0 d, 1 d, 2 d and 3 d. Data are presented as mean ± SD, *n* = 3 biological replicates. ns, not significant (*p* > 0.05), * *p* < 0.05, ** *p* < 0.01, *** *p* < 0.001; Student’s *t*-test

An ideal controlling system should be capable of quickly responding to both ON and OFF signals as required. To demonstrate how the transcriptional regulatory activities of iCRISPRa/i systems respond to 4OHT, we evaluated the 4OHT responsiveness by the transcriptional activation of target gene and protein subcellular localization analysis. Twenty-four hours after transfection of iCRISPRa and sgKlf4 plasmids, transfected cells were treated with either ethanol or 1 µM 4OHT, and the transcriptional levels of *Klf4* were quantified at 0 h, 4 h, 8 h, 12 h and 24 h by qPCR. Compared with the transcriptional level of *Klf4* detected at 0 h, the transcriptional activation of *Klf4* was observed after 4OHT induction for 4 h relative to ethanol-treated cells and plateaued at 8 h, and further treatment did not increase the transcriptional activation (Fig. 5a). Based on the above experimental results, HEK 293T cells were transfected with iCRISPRa plasmid and non-treated or treated with either ethanol or 1 µM 4OHT for 4 h, and the protein subcellular distribution was detected by Western blot. With non-treatment (0 h) or ethanol treatment for 4 h, iCRISPRa proteins were less nuclear-localized. Upon 4 h of 4OHT treatment, the proportion of nuclear-localized iCRISPRa protein increased significantly to 58.03% (Fig. 5b), comparable to that with 4OHT treatment for 24 h (Fig. 1c). Similar trends were also observed in the iCRISPRi system. After 4 h of 4OHT treatment, iCRISPRi could achieve remarkable transcriptional repression on the *Prdm4* transcript, and the transcriptional repression was stabilized by persistent 4OHT induction (Fig. 5c). In the subcellular localization analysis, iCRISPRi fusion protein was less nuclear-localized with non-treatment (0 h) or ethanol treatment for 4 h; upon 4 h of 4OHT treatment, the proportion of nuclear-localized iCRISPRi protein increased significantly to 60.78% (Fig. 5d), comparable to that with 4OHT treatment for 24 h (Fig. 1e). These results suggest that iCRISPRa/i proteins effectively regulated transcription with 4OHT treatment through rapidly translocating from cytoplasm to nucleus.

To demonstrate whether the regulatory activities of the iCRISPRa/i systems were reversible, we first explored the reversibility of the iCRISPRa system on the transcriptional activation of *Klf4* and subcellular localization of iCRISPRa protein. HEK 293T cells were transfected with iCRISPRa plasmid alone or co-transfected with sgRNA plasmid targeting *Klf4* transcript and treated with 4OHT to induce iCRISPRa nuclear localization. After induction, 4OHT was replaced by ethanol, and the transcript and protein distribution were detected at different time points. After 4OHT retraction for 0 d, the transcriptional level of *Klf4* showed significant activation. However, shortly after 4OHT withdrawal, the altered expression of *Klf4* started to be reversed and gradually restored to the levels of non-inducible status with the extension of time of 4OHT retraction (Fig. 5e). The subcellular localization of iCRISPRa showed that after 4OHT retraction for 2 d, the percentage of nuclear-localized iCRISPRa protein was decreased; with prolonged 4OHT retraction for 3 d, a lower percentage of nuclear-localized iCRISPRa protein was observed (Fig. 5f), similar to that without 4OHT treatment (Fig. 1c). Similar to the iCRISPRa system, the transcriptional repression of *Prdm4*, target gene of iCRISPRi, was reversed and gradually restored to the levels of non-inducible status with the extension of time of 4OHT retraction (Fig. 5g). Meanwhile, iCRISPRi protein showed a significant reduction of the percentage of nuclear-localized protein after 4OHT retraction for only 1 d (Fig. 5h), similar to that without 4OHT treatment (Fig. 1e), demonstrating a more sensitive reversibility. These data indicated that iCRISPRa/i systems were successfully switched on upon 4OHT induction and switched off with 4OHT retraction within a short period. Hence, our results suggest that the activities of iCRISPRa/i systems were reversible and showed exceptional “on-off” kinetics.

Benchmarking with Dox-inducible CRISPRa/i systems, iCRISPRa/i had a higher response efficiency and a comparable invertibility. Unlike the majority of iCRISPRa/i proteins could be transported from the cytoplasm to the nucleus within 4 h 4OHT treatment which was similar to that of 24 h 4OHT treatment, with doxycycline treatment for 4-8 h, the expression of TRE-CRISPRa/i proteins has been initiated and reached the highest in 12 h (Fig. S10a and b). In terms of regulation reversibility, both of iCRISPRa/i and TRE-CRISPRa/i could be restored to their non-inducible state within 3 d and remain a low background (Fig. S10c and d). These data showed that iCRISPRa/i systems respond to inducer faster and regulate the transcription of target genes in a narrower time window, compared with Dox-inducible TRE-CRISPRa/i systems.

### iCRISPRa/i altered tyrosinase activity and melanin content by regulating tyrosinase gene transcription with 4OHT induction

To validate whether our iCRISPRa/i systems could induce phenotypic changes by controlling endogenous gene expression, we set to regulate tyrosinase (*Tyr*) gene transcription in the B16 melanoma cells. *Tyr* gene encodes TYR protein, the rate-limiting enzyme for melanogenesis [45–48]. Previous reports have shown that up-regulated *Tyr* transcription is related to increased melanin production [49], while knockout of tyrosinase caused pigment loss in melanoma cells [50], zebrafishes [17], mice [51–54] and rabbits [55]. We first explored whether altering the *Tyr* transcript could change tyrosinase activity and melanin content using non-inducible CRISPRa/i. B16 melanoma cells were transfected with non-inducible CRISPRa/i plasmids co-expressing with sgRNA and GFP reporter (Fig. S11a). The plasmid carrying a puromycin resistance gene was co-transfected to enrich the transfected cells through puromycin selection. Transfected cells were cultured with RPMI 1640 media containing 10% FBS until reaching 80% confluence, then the media were replaced by the DMEM media containing 10% FBS to induce melanin synthesis. *Tyr* transcript was significantly upregulated along with the prominent increase of tyrosinase activity and melanin content by co-expressing CRISPRa and sgTyr-a (Fig. S11b-d). Meanwhile, *Tyr* transcription also could be repressed and the tyrosinase activity and melanin content were reduced correspondingly by the expression of CRISPRi and sgTyr-i (Fig. S11e-g).

**Fig. 6:**
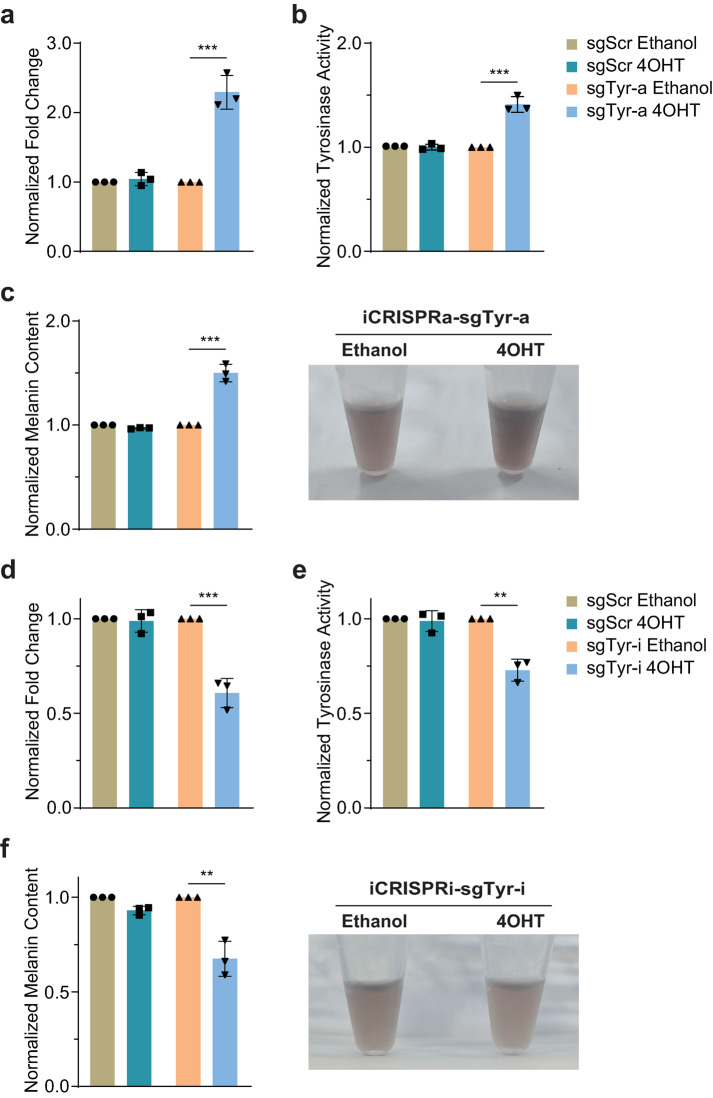
iCRISPRa/i altered tyrosinase activity and melanin content by regulating tyrosinase gene transcription with 4OHT induction. **a-c** The *Tyr* transcript **a**, tyrosinase activity **b** and melanin content **c** of B16 cells infected with iCRISPRa and sgRNA lentiviruses were analyzed after ethanol or 4OHT treatment. **d-f** The *Tyr* transcript **d**, tyrosinase activity **e** and melanin content **f** of B16 cells infected with iCRISPRi and sgRNA lentiviruses were analyzed after either ethanol or 4OHT treatment. Transcriptional levels of *Tyr* of each sample were detected by qPCR and normalized to the housekeeping gene *Gapdh*. Tyrosinase activities were determined by L-DOPA oxidation. Melanin contents were measured by a Mexameter. Data are presented as mean ± SD, *n* = 3 biological replicates. ** *p* < 0.01, *** *p* < 0.001; Student’s *t*-test

Next, to investigate the effects of iCRISPRa/i on *Tyr* transcript, tyrosinase activity and melanin content, B16 cells were co-infected with iCRISPRa/i and sgRNA lentiviruses to generate stable B16-iCRISPRa/i-sgRNA cell lines by the antibiotic selection, then the transcriptional levels of *Tyr* were detected after either ethanol and 4OHT treatment by qPCR. B16 cells infected with iCRISPRa/i and sgScr lentiviruses had no significant difference in *Tyr* transcription either treated with ethanol or 4OHT. Furthermore, there were no obvious changes in tyrosinase activity and melanin content under these conditions (Fig. 6a-f). However, the *Tyr* transcription, tyrosinase activity and melanin content responded to 4OHT induction in B16-iCRISPRa/i-sgTyr-a/i cell lines. For iCRISPRa, the transcriptional level of *Tyr* had an approximately 2.29-fold increase with 4OHT induction, accompanied by notable increases of tyrosinase activity and melanin content in the presence of 4OHT, 1.41-fold and 1.50-fold, respectively (Fig. 6a-c). Similarly, iCRISPRi repressed *Tyr* transcription and resulted in reduced tyrosinase activity and melanin content upon 4OHT induction (Fig. 6d-f). These results suggested that iCRISPRa/i systems could change tyrosinase activity and melanin content of melanocytes by altering tyrosinase gene transcription in the presence of 4OHT.

### Fusion deficit of C2C12 cells resulted from inducible *Mymx* repression using iCRISPRi system

Next, we took a rigorous test to verify whether our iCRISPRi system could induce transcriptional repression and corresponding phenotypic change in C2C12 myoblasts. We chose *Myomixer* (*Mymx*) as our target gene. *Mymx* encodes the protein Myomixer, also named Myomerger or Minion, which plays an essential role in myoblast fusion by forming a two-component membrane fusion complex with Myomaker [56]. Disruption of *Mymx* prevented the fusion of C2C12 myoblasts [57], and deletion of *Mymx* in satellite cells abolished satellite cell fusion and prevented muscle regeneration in transgenic mice [58]. We first examined the endogenous expression pattern of *Mymx* and found that the transcription of *Mymx* increased sharply 2 d after differentiation and remained moderately increasing in subsequent induction (Fig. S12a), which was similar to other myogenic and myoblast fusion factors, such as *Myogenin*, *Myf6* and *Myomaker* [59, 60].

We then investigated whether a down-regulated *Mymx* transcript by non-inducible CRISPRi could influence the fusion of myoblasts. C2C12 myoblasts were transfected with CRISPRi plasmids co-expressed with sgRNA and GFP reporter and switched to fusion media for 4 d. Subsequently, cells were fixed and stained with antibodies against Desmin, a muscle-specific class III intermediate filament, and DAPI (Fig. S12b). As shown in Fig. S12c-g, the immunofluorescence and quantitative analyses showed that CRISPRi inhibits myoblast fusion into myotubes under the guidance of sgMymx.

**Fig. 7:**
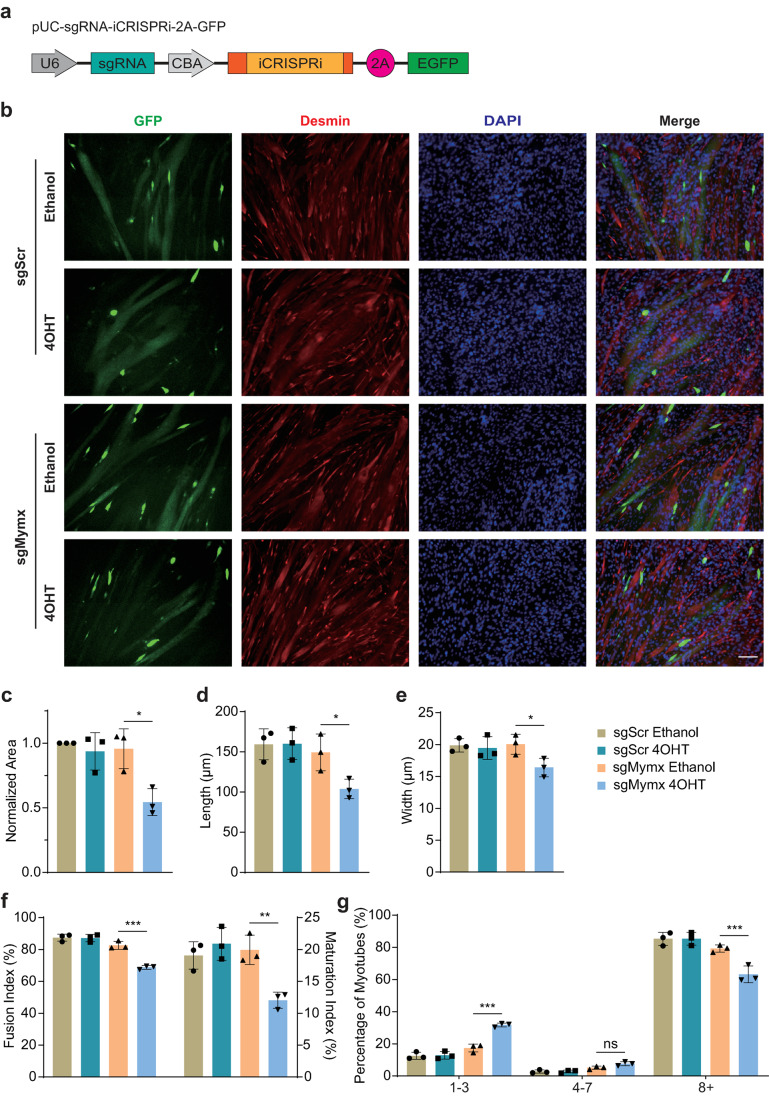
Fusion deficit of C2C12 cells resulted from inducible *Mymx* repression using iCRISPRi system. **a** Schematic of pUC-sgRNA-iCRISPRi-2A-GFP. sgRNA was driven by the U6 promoter, iCRISPRi and GFP were driven by the chicken β-actin (CBA) promoter and split by a self-cleaving 2A peptide. **b** Immunofluorescence images of differentiated C2C12 cells in which fusion of myoblasts was inhibited by iCRISPRi with 4OHT induction. The transfected C2C12 myoblasts were incubated in the fusion media containing either ethanol or 1 µM 4OHT for 2 d. After an additional two-day period of fresh fusion media, differentiated cells were fixed and stained with antibodies against Desmin, and cell nuclei were stained with DAPI. Scale bar, 100 μm. All photographs shown in this figure are representative of three independent experiments. **c-e** Quantification of the normalized area **c**, length **d** and width **e** of GFP-positive cells. **f**,** g** Quantification of the fusion and maturation index **f** and the percentage of cells with 1-3, 4-7 and 8+ nuclei **g**. Data are presented as mean ± SD, *n* = 3 biological replicates. ns, not significant (*p* > 0.05), * *p* < 0.05, ** *p* < 0.01, *** *p* < 0.001; Student’s *t*-test

Using the iCRISPRi system, we investigated whether the inducible downregulation of *Mymx* expression could alter myoblasts formation similarly to CRISPRi. C2C12 myoblasts were transfected with iCRISPRi plasmids which co-expressed sgRNA and GFP reporter (Fig. 7a), and subsequently cultured in fusion media supplemented with either ethanol or 4OHT for 2 d. Following induction, cells were switched to fresh fusion media for two more days. Fixed C2C12 cells were immunostained using antibodies against Desmin and counterstained with DAPI (Fig. 7b). Our results demonstrated that regardless of treatment conditions, no significant differences in myoblast differentiation were observed among cells transfected with iCRISPRi and sgScr. However, compared to ethanol treatment, 4OHT induction markedly inhibited the differentiation of cells transfected with iCRISPRi and sgMymx. Upon 4OHT induction, the area of GFP-positive cells exhibited a reduction of 44.56%, while the length and width showed reductions of 29.39% and 18.22%, respectively (Fig. 7c-e). These results were further supported by the quantification of the fusion index, maturation index and the percentage of cells with 1-3, 4-7 and 8+ nuclei in the total GFP-positive cells. Upon 4OHT induction, the cells transfected with iCRISPRi and sgMymx showed a decrease of 16.82% in fusion index and a decrease of 39.64% in maturation index along with an average increase of 82.69% in cells containing only 1-3 nuclei and an average reduction of 21.13% in cells containing 8+ nuclei, compared with those without 4OHT induction (Fig. 7f and g). These findings suggest that repression of *Mymx* transcription in C2C12 myoblasts mediated by iCRISPRi and sgMymx upon 4OHT induction resulted in anticipated phenotypic alterations, indicating that iCRISPRi system not only effectively suppresses target transcript expression but also induces specific cellular phenotypic changes.

## Discussion

In this study, we have developed a dCas9-based transcriptional regulation strategy, iCRISPRa/i, that allows tight temporal control of transcriptional regulation using a drug input. The ERT2 domains effectively sequester fusion proteins outside the nucleus to prevent them from performing transcriptional regulatory activities. However, the fusion proteins translocate rapidly into the nucleus in the presence of 4OHT. Once 4OHT is withdrawn, the fusion proteins are relocated to the cytoplasm and the original levels of transcription of target genes are restored. The efficiencies of gene expression regulation of iCRISPRa/i were comparable to those of non-inducible and doxycycline-inducible counterparts. The iCRISPRa/i systems also outperform TRE-CRISPRa/i by achieving tighter control of basal gene expression and more rapid drug-inducible transcriptional regulation. The phenotypes also could be changed through transcriptional regulation by iCRISPRa/i with 4OHT induction. Thus, we have established a rapid and reversible transcriptional regulatory system useful for inducing phenotypic change in cultured cells.

Gene expression manipulation is indispensable to studying the function of genes of interest, and the emergence of CRISPR/Cas9 and CRISPRa/i systems made control of gene expression simple, rapid and convenient. Limited by various shortcomings, an inducible gene expression manipulation is urgently needed. Multiple designs of inducible manipulation systems have been developed in previous studies. Basically, the inducibility can be achieved through the regulation of Cas9/dCas9 or sgRNA. For instance, the expression of Cas9/dCas9 could be under the control of doxycycline or heat-shock-responsive promoters [9–11, 17], or the activity of the dCas9 could be regulated by the site-specific installation of photocaged lysine amino acids or heterodimerization of CRY2 and CIB1 fused to functional domains [12, 13]. Photocleavable protectors or caged nucleobases were introduced [14, 15] to regulate the expression of sgRNA. Even more sophisticated, a chemically inducible sgRNA is achieved by removing covalent chemical groups [16]. However, these approaches have inherent limitations in practice. The doxycycline-inducible system takes effect slowly and transcriptional repression persists even after doxycycline withdrawal for several days [9, 10]. The application of heat-inducible systems is hindered by the rather limited treatment durations. Other approaches also face technical challenges in the preparation of modified Cas9 or sgRNAs. Hence, more practically inducible CRISPRa/i systems are worthy of consideration to achieve rapid, effective and reversible gene expression manipulation.

During embryonic development and physiologic processes, gene expressions are under stringent control to perform their functions precisely in a certain time window. As pointed out by Waddington [61, 62], cell state transition could be achieved by activation of genes at the key time points and the cells would follow the transition path promptly even without prolonged activation of the genes. Conventionally, one cell type could be reprogrammed into pluripotent stem cells and other cell types using continuous ectopic expression of transcription factors [63–66]. The lack of temporal resolution in this approach makes understanding the fine-tuned underlying mechanisms hardly possible. Using iCRISPRa/i systems introduced here, transcriptional regulation could be achieved shortly after the inducement of 4OHT, and inducible transcriptional regulation was reversed upon 4OHT withdrawal. We could utilize the iCRISPRa/i systems to explore the key time point of transcriptional control in the development and cell transdifferentiation and to tackle the key questions in the mechanisms of cell fate determination and chromosome structure change in the development.

A major challenge in the application of CRISPR technology is the off-target probability of the Cas9/dCas9. Different strategies have been developed to reduce the off-target possibility of Cas9/dCas9: optimizing the design strategies of sgRNA [67], using paired nCas9 or dCas9-FokI [68, 69], improving the specificity of Cas9/dCas9 by structure-guided engineering [70] and decreasing the dosage or the active time of Cas9/dCas9 [71] within the cells. Limiting the dosage or the active time of the active enzyme could be achieved by initiating gene transcription under the control of the TRE or heat-shock promoters [9, 17] or eliminating photoprotective groups to restore Cas9 activity with light [12]. Compared with other inducible CRISPRa/i systems, drug-inducible CRISPRa/i systems have more advantages, such as fewer technical challenges, more flexible induction time and particularly the more flexible “on-off” kinetics, which could further improve the targeting specificity. Different from Dox-inducible TRE-CRISPRa/i systems, iCRISPRa/i proteins were transported from the cytoplasm into the nucleus to regulate gene transcription with 4OHT induction in a shorter induction duration. In our study, both of them could restore to a non-inducible state in a similar retracement duration, suggesting there may be no difference in off-target activity. However, dysregulated genes could be generated by iCRISPRa/i and TRE-CRISPRa/i systems due to the side effects of inducers. After doxycycline treatment, mitochondrial proteostasis and function were disturbed through downregulated genes involved in mitochondrial transport, mitochondrial protein synthesis, mitochondrial membrane potential, ATP synthesis and electron transport chain [72]. Damaged mitochondria would promote inflammatory response through releasing damage-associated molecular patterns [73]. As an estrogen analogue, tamoxifen competitively binds to ERs and reduces estrogen-induced effects [43]. These studies suggest that the use of Dox and 4OHT in experimental models may introduce confounding variables into study outcomes. Therefore, researchers should judiciously select these agents based on their specific methodological requirements.

Previous research showed that strong transcriptional regulatory activity was obtained by targeting CRISPRa/i to windows of DNA from -400 to -50 bp and -50 to +300 bp relative to the transcription start site of target genes, respectively [9]. The increase in amino acid quantity may change the spatial structure of protein, so iCRISPRa/i which fuses with ERT2 domains may have a different space structure and altered targeting DNA windows, compared with CRISPRa/i proteins. In our study, the transcriptional activation activity mediated by iCRISPRa with 4OHT induction was obtained with sgRNAs in a DNA window from -300 to +130 bp; and the transcriptional repression activity of iCRISPRi was obtained with sgRNAs in a DNA window from +20 to +130 bp, except sgMstn. These DNA windows of sgRNA guiding iCRISPRa/i to target sites highly overlapped with those used for CRISPRa/i. Furthermore, compared to CRISPRa/i, iCRISPRa/i systems exhibited similar efficiency in transcriptional regulation when employing the same sgRNA, suggesting that fusions of ERT2 domains to CRISPRa/i do not affect the capacity of activation and repression, but isolating fusion protein from nucleus without 4OHT induction and transporting from cytoplasm to nucleus upon 4OHT stimulation.

We have determined that variable activation and repression activities can be achieved with different dosages of 4OHT. Previous studies have reported that dCas9 allied with multiple potent transcription activators could achieve high-level transcriptional activation, up to 1,000-4,000 folds [74]. However, lower levels of transcriptional activation at physiological conditions are sufficient to induce cellular transition into another state. For instance, a modest transcriptional activation of *Ngn2* and *Isl1* by only 5-10 folds can drive astrocytes transdifferentiation into neurons [75]. Moreover, higher expression up-regulation than physiologic level could be toxic, as evidenced in the gene therapy for neurodegenerative disease spinal muscular atrophy (SMA), in which case, overexpression of a copy of “healthy” gene (encoding survival motor neuron protein) disturbs RNA manipulation and results in SMA-like pathogenic events by gain-of-function mechanisms [76], highlighting the importance of dose-dependent transcriptional regulation. Therefore, iCRISPRa/i systems are more attractive due to their precise transcriptional regulation achieved by varying dosages of 4OHT.

In conclusion, we have developed a conditional transcriptional regulatory system that is rapidly inducible, targeting multiple gene sites simultaneously and reversible. The fast response time will enable applications that demand tight temporal control. Hence, our ERT2-based iCRISPRa/i technology expands the toolkit for precise transcriptional regulation in mammalian cells.

## Supplementary Information

Below is the link to the electronic supplementary material.

Supplementary file1 Supplementary Fig. S1-12 (PDF 28.5 MB)

Supplementary file2 Tables S1-3 and Supplementary Figure Legends (DOCX 36.8 KB)

Supplementary file3 Supplementary Data 1. Constructs sequence (DOCX 25.6 KB).

Supplementary file4 Supplementary Data 2. DEG_iCRISPRa (XLSX 28.2 KB)

Supplementary file5 Supplementary Data 3. DEG_iCRISPRi (XLSX 42.9 KB)

Supplementary file6 Supplementary Data 4. DEG of iCRISPRa_GOenrich (XLSX 18.3 KB)

Supplementary file7 Supplementary Data 5. DEG of iCRISPRi_GOenrich (XLSX 18.6 KB)

Supplementary file8 Supplementary Data 6. Numerical Data (XLSX 44.9 KB)

## Data Availability

The raw sequence data reported in this paper have been deposited in the Genome Sequence Archive [77] in National Genomics Data Center [78], China National Center for Bioinformation/Beijing Institute of Genomics, Chinese Academy of Sciences (GSA-Human: HRA010719 for iCRISPRa and HRA010718 for iCRISPRi) that are publicly accessible at https://ngdc.cncb.ac.cn/gsa-human. The numerical data that underlies the graphs is provided in Supplementary Data 6.

## Materials availability

The sequences of CRISPRa/i, TRE-CRISPRa/i and iCRISPRa/i constructs used in this study are listed in Supplementary Data 1.
