# Correction: Novel drug-inducible CRISPRa/i systems for rapid and reversible manipulation of gene transcription

**DOI:** 10.1007/s00018-025-05914-3

**Published:** 2025-11-14

**Authors:** Ming Sui, Meiling Zhou, Mengge Cui, Huan Liu, Xiaolin Zhang, Na Hu, Yang Li, Beibei Wang, Guojun Yang, Pengling Gui, Lingqiang Zhu, Feng Wan, Bin Zhang

**Affiliations:** 1 Department of Physiology, School of Basic Medicine, Tongji Medical College, Huazhong University of Science and Technology, Wuhan, 430030 China; 2 The Institute for Brain Research, Collaborative Innovation Center for Brain Science, Huazhong University of Science and Technology, Wuhan, 430030 China; 3 Hubei Key Laboratory of Drug Target Research and Pharmacodynamic Evaluation, Huazhong University of Science and Technology, Wuhan, 430030 China; 4 Key Laboratory of Hubei Province for Cognitive and Affective Disorders, School of Medicine, Wuhan Institute of Biomedical Sciences, Jianghan University, Wuhan, 430056 China; 5 Department of Clinical Laboratory, The 980Th Hospital of PLA Joint Logistical Support Force (Bethune International Peace Hospital), Shijiazhuang, 050000 China; 6 Department of Neurosurgery, Guangdong Provincial People’s Hospital (Guangdong Academy of Medical Sciences), Southern Medical University, Guangzhou, 510080 China; 7 Department of Blood Transfusion, Zhongnan Hospital of Wuhan University, Wuhan, 430071 China; 8 School of Medicine, Hebei University of Engineering, Handan, 056038 China; 9 Department of Pathophysiology, School of Basic Medicine, Tongji Medical College, Huazhong University of Science and Technology, Wuhan, 430030 China

**Correction: Cellular and Molecular Life Sciences (2025) 82:249**

10.1007/s00018-025-05786-7

In this article Figs. 1, 2, 3, 4, 5, 6 and 7 appeared incorrectly and have now been corrected in the original publication. For completeness and transparency, the old incorrect versions are displayed below.

Fig 1

Incorrect version
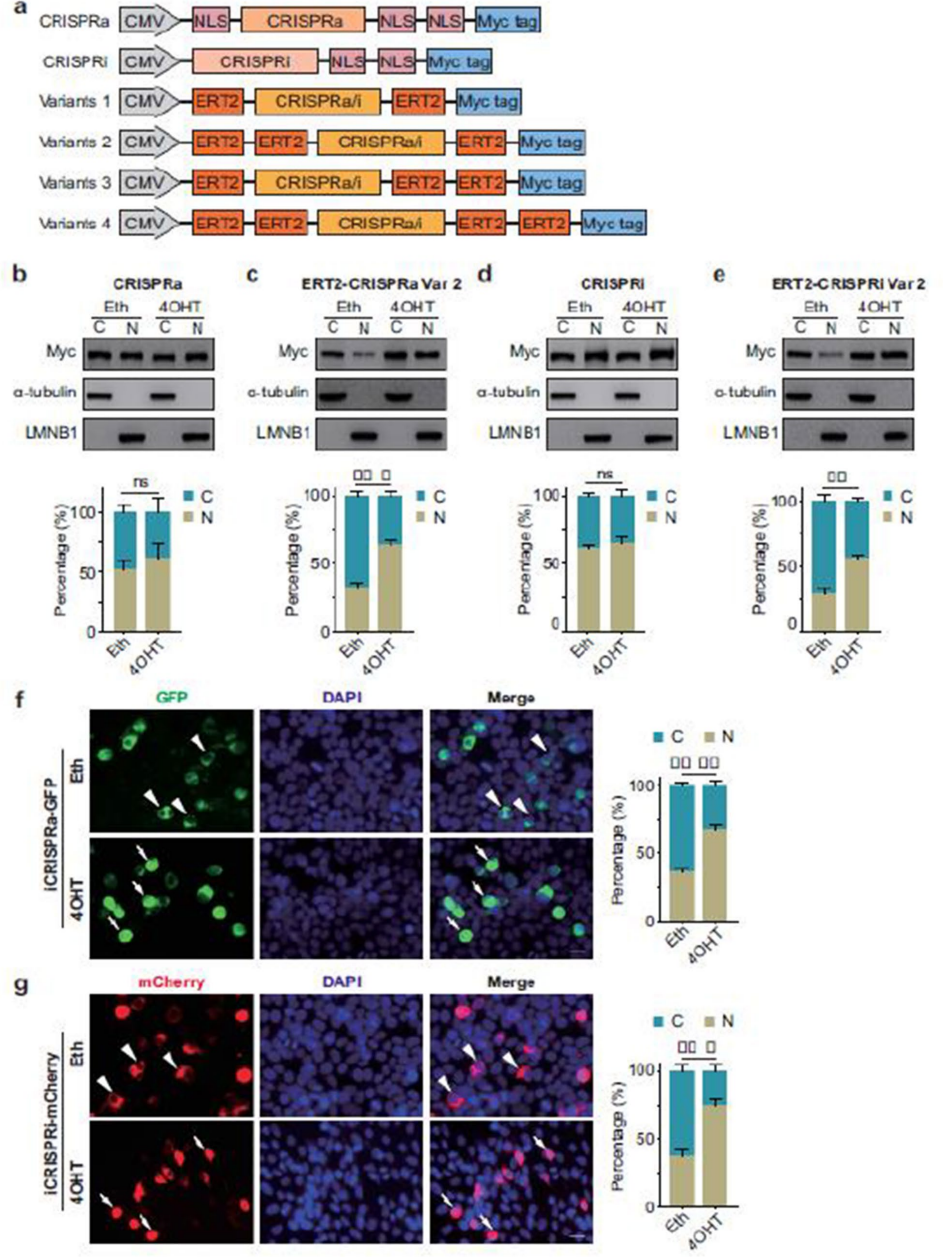


Corrected version > 
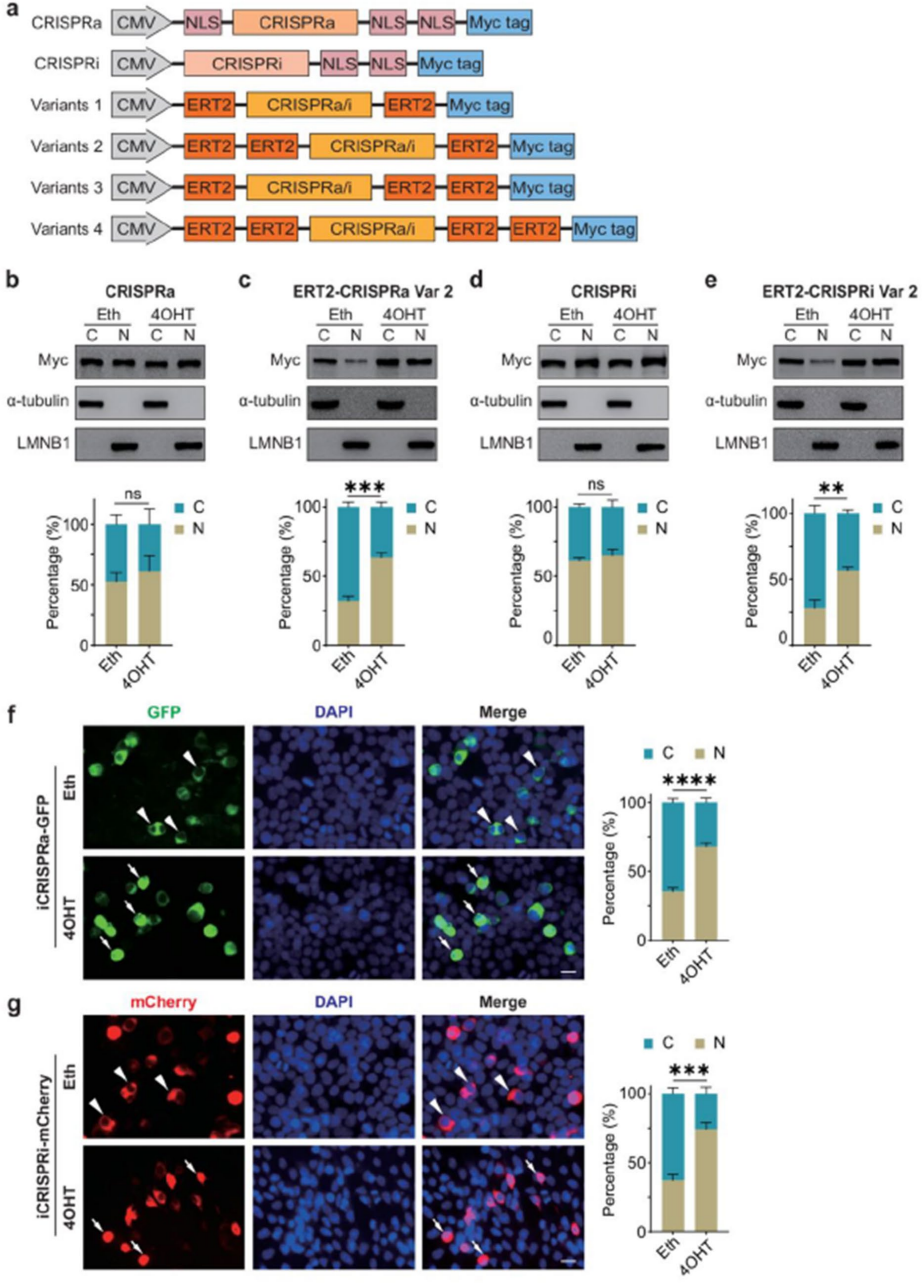


Fig 2

Incorrect version
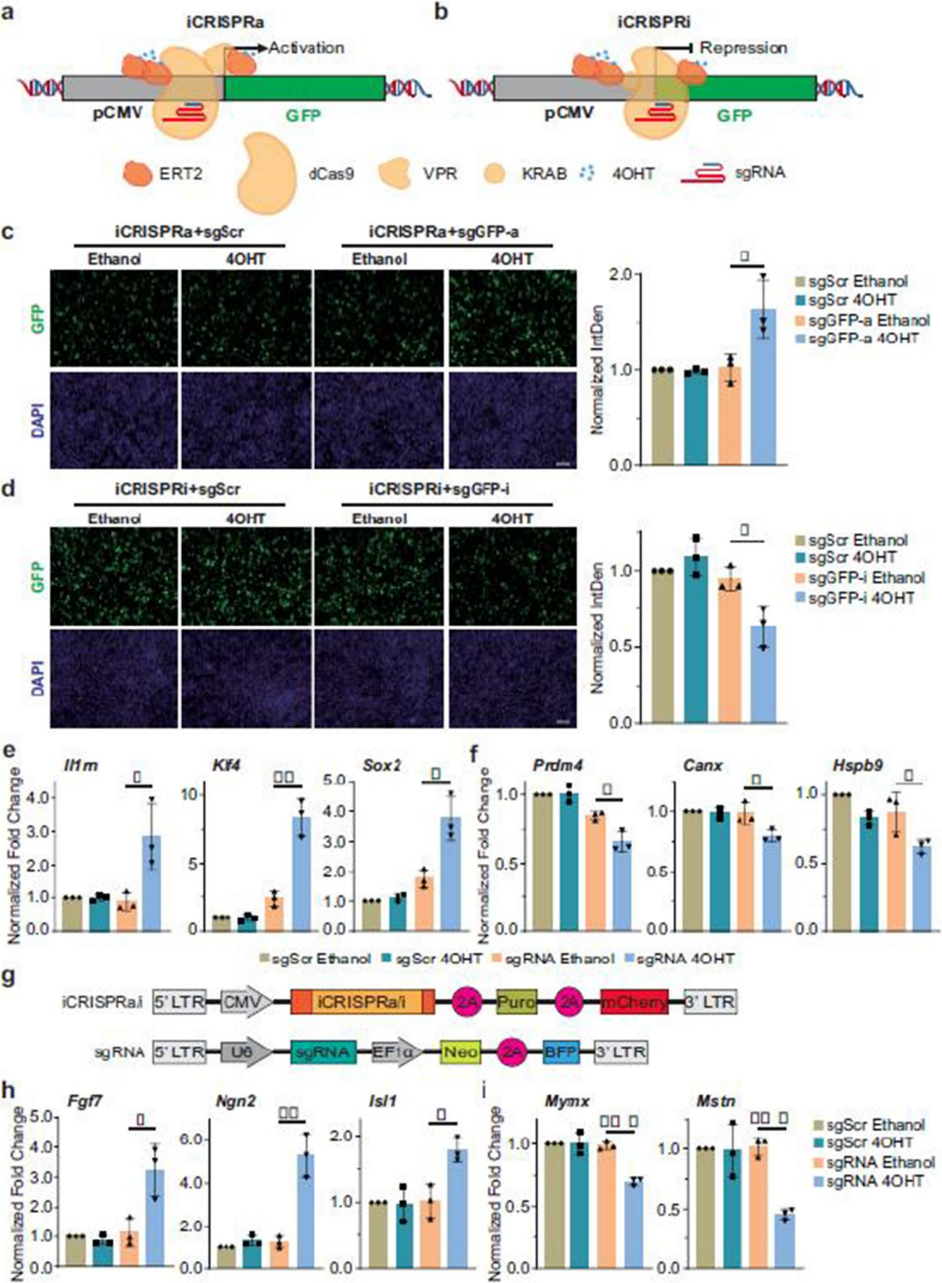


Corrected version
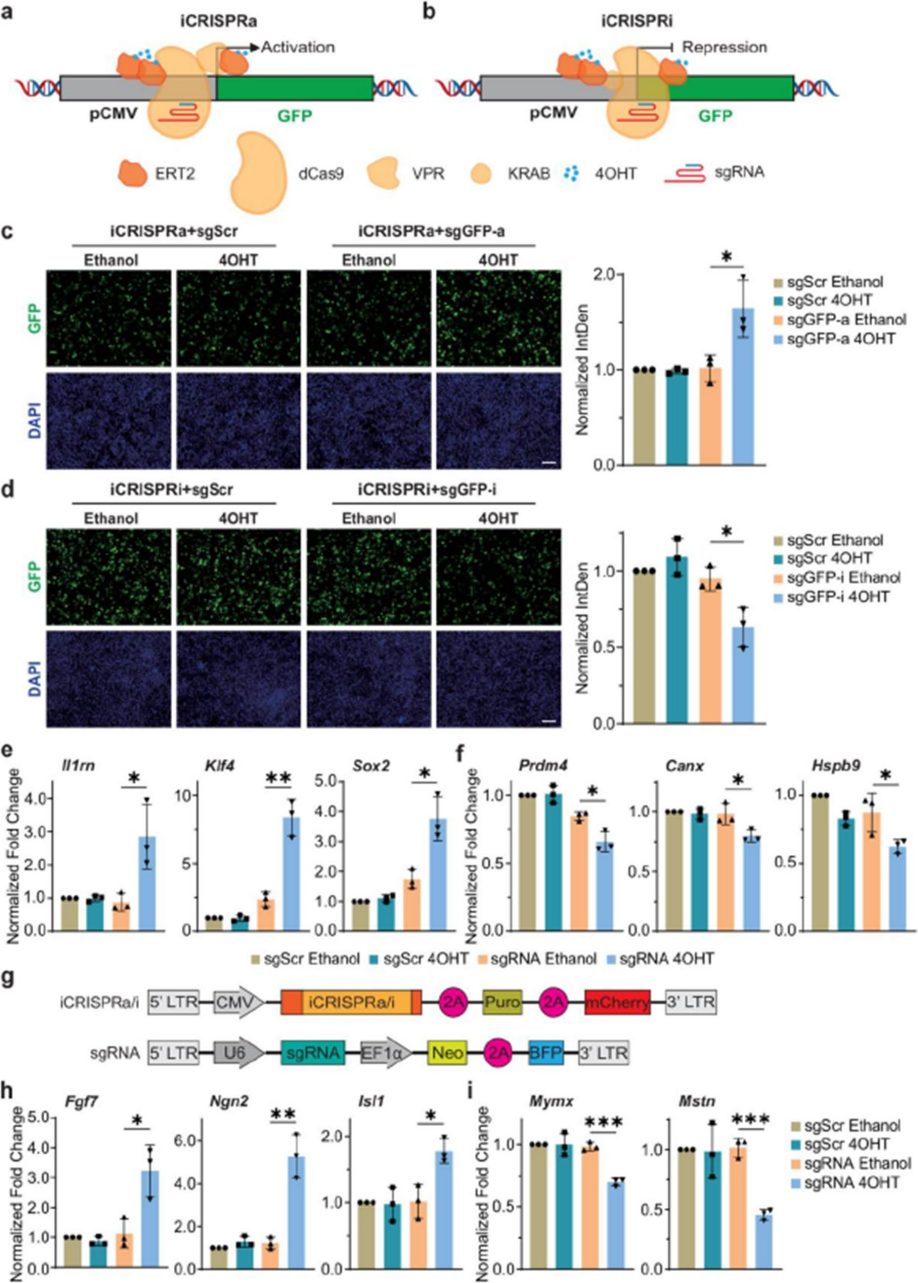


Fig 3

Incorrect version
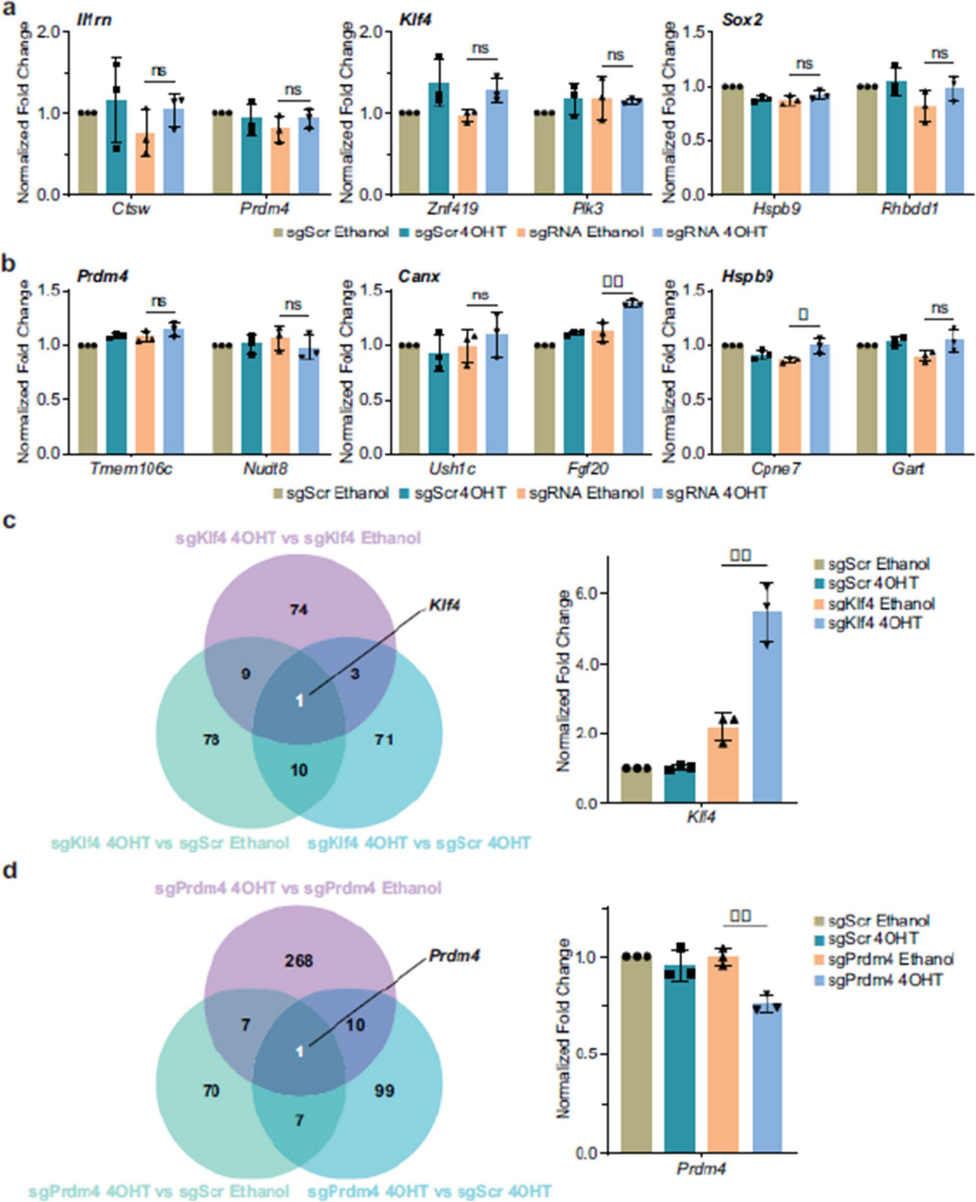


Corrected version
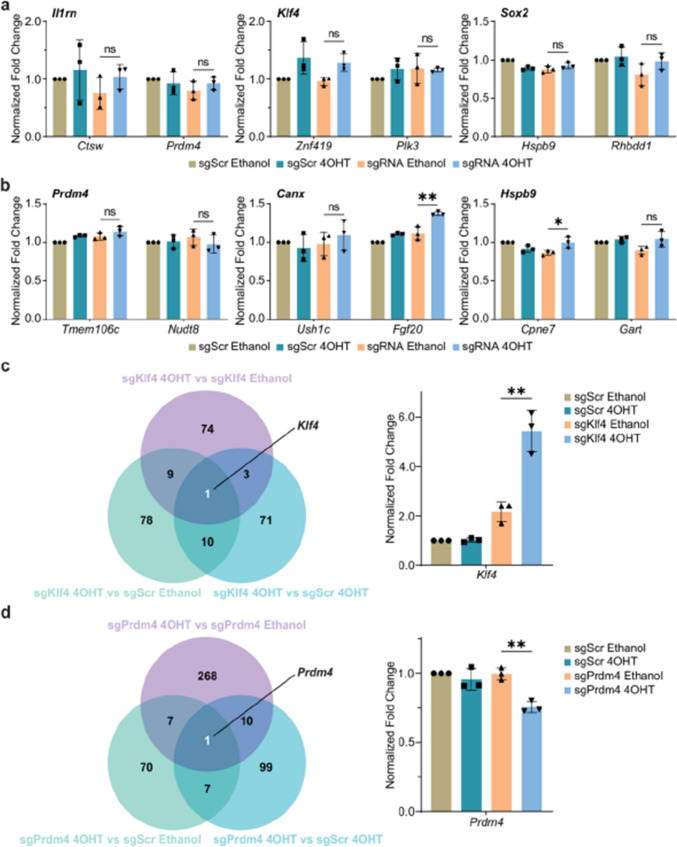


Fig 4

Incorrect version
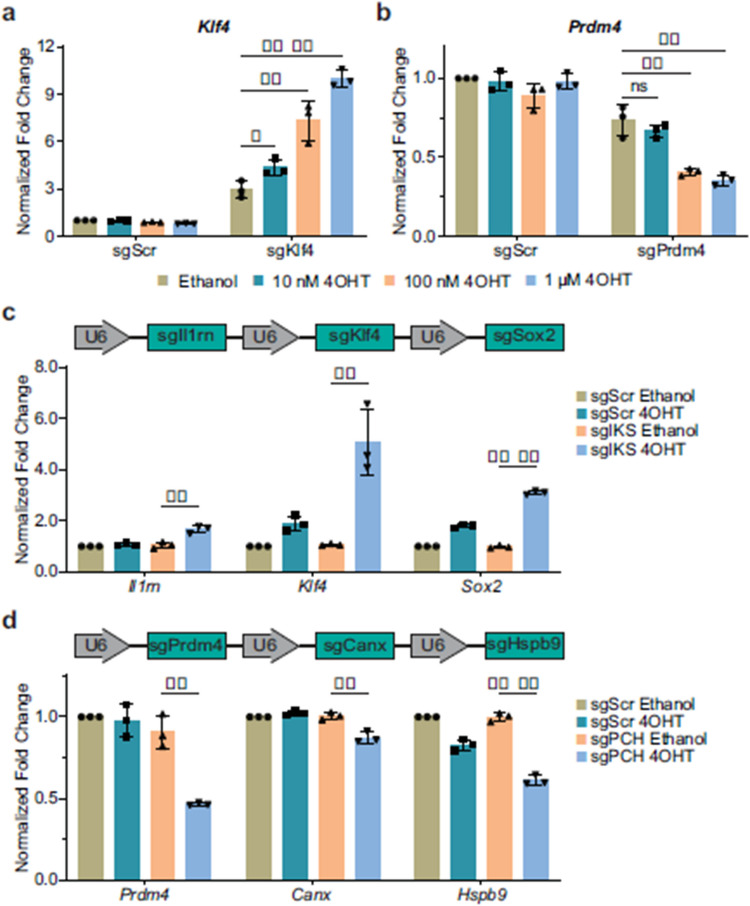


Corrected version
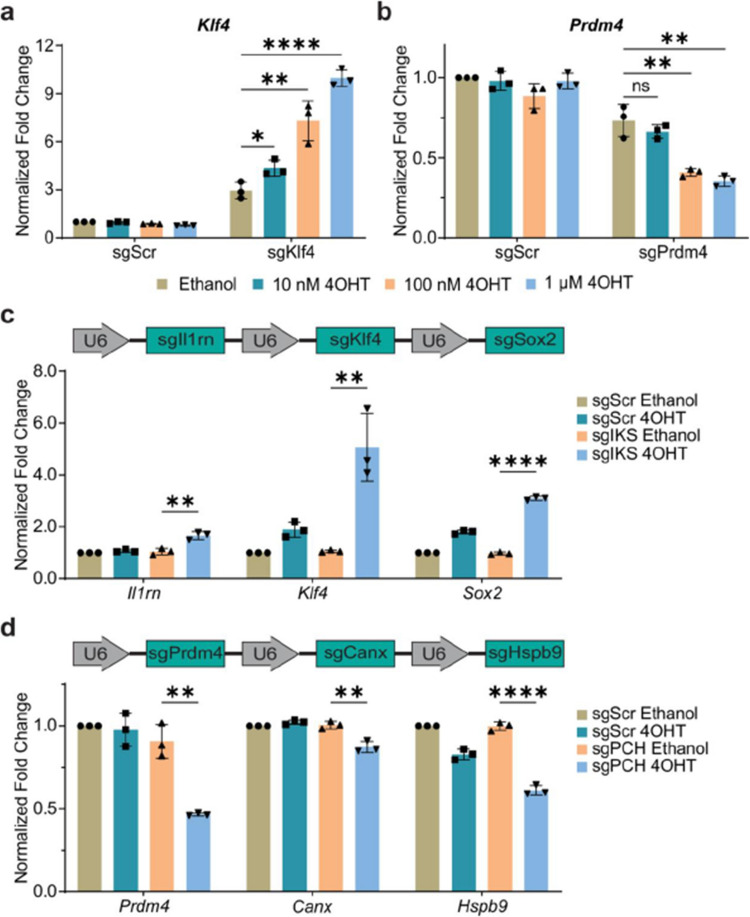


Fig 5

Incorrect version
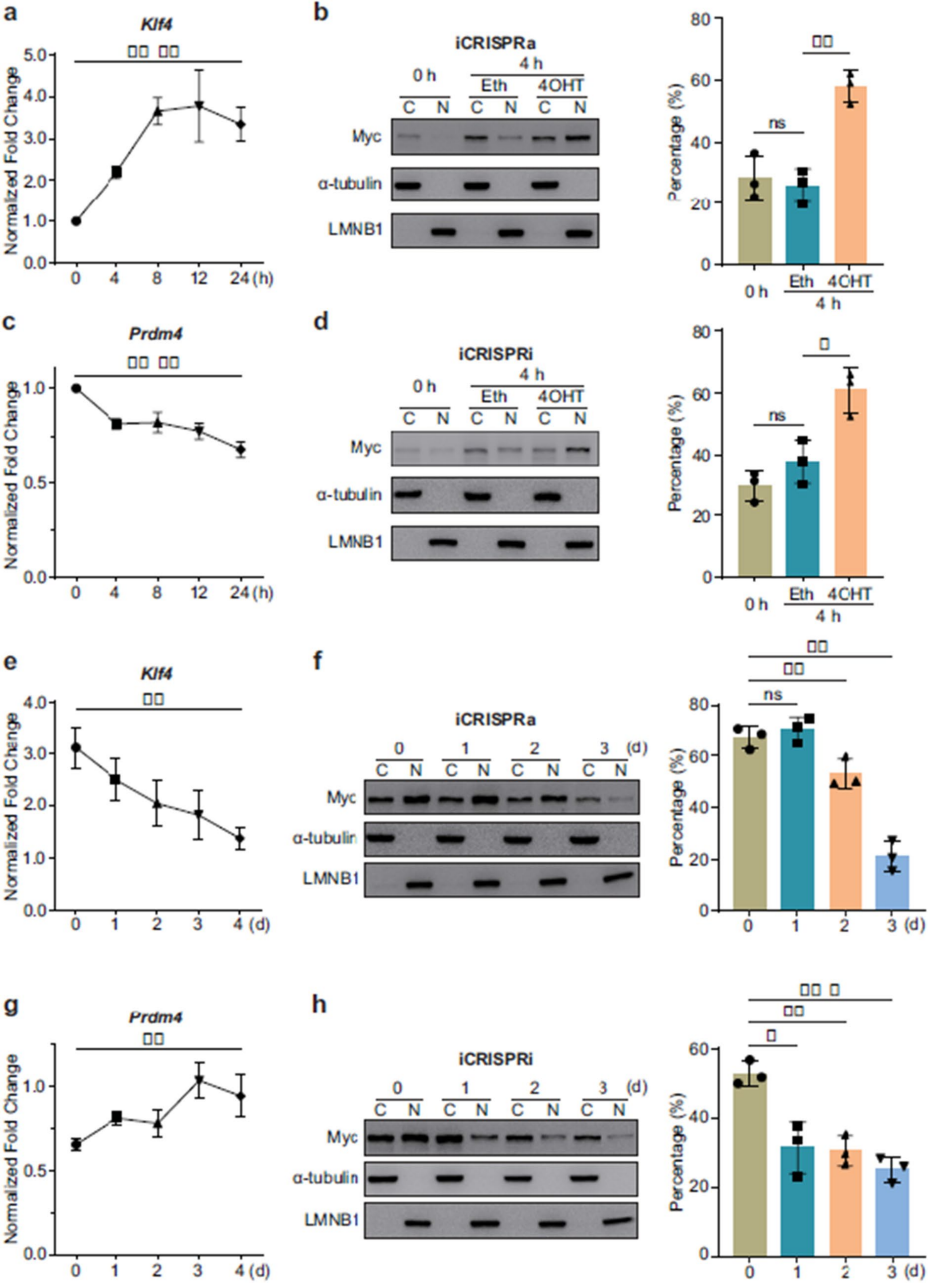


Corrected version
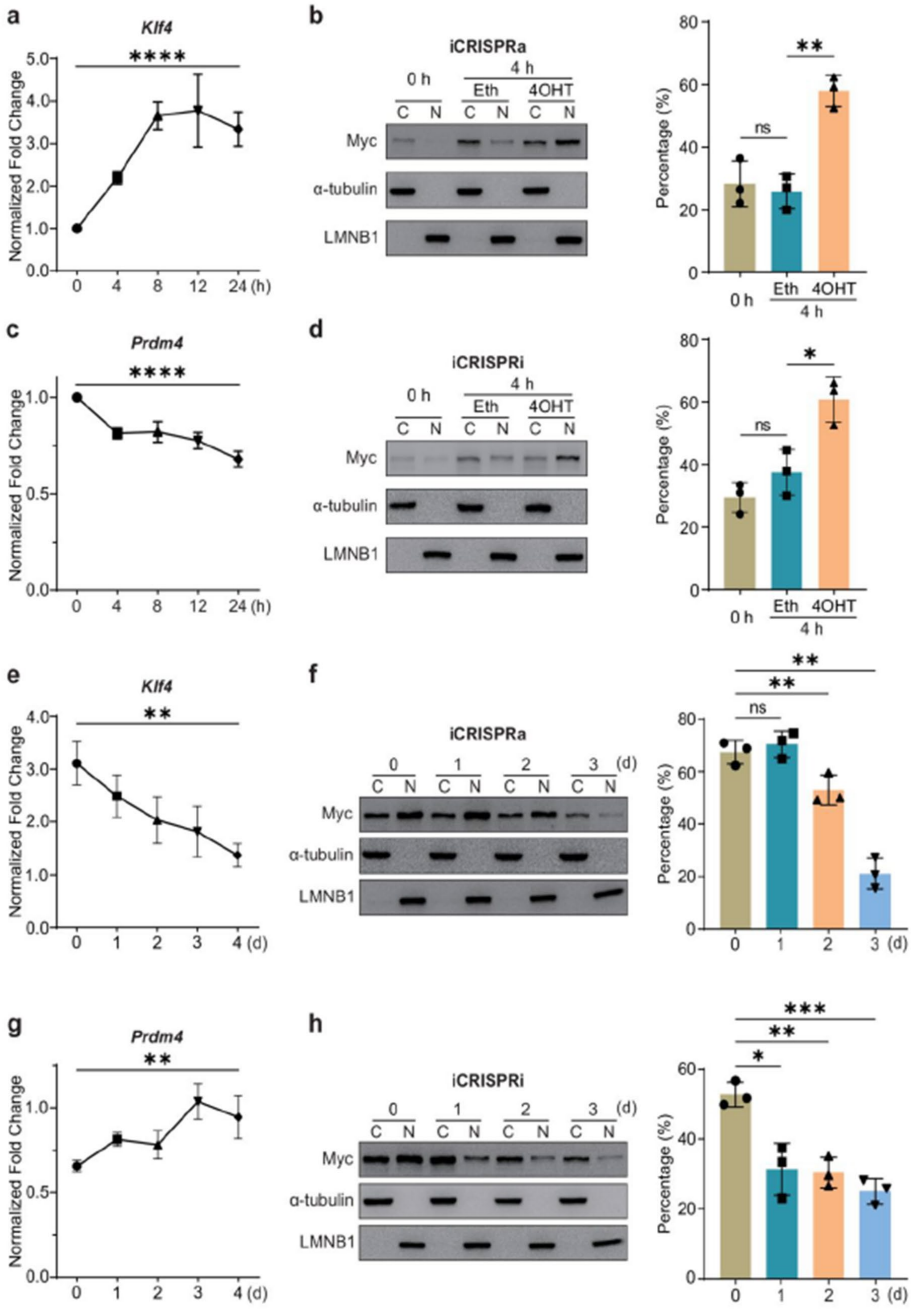


Fig 6

Incorrect version
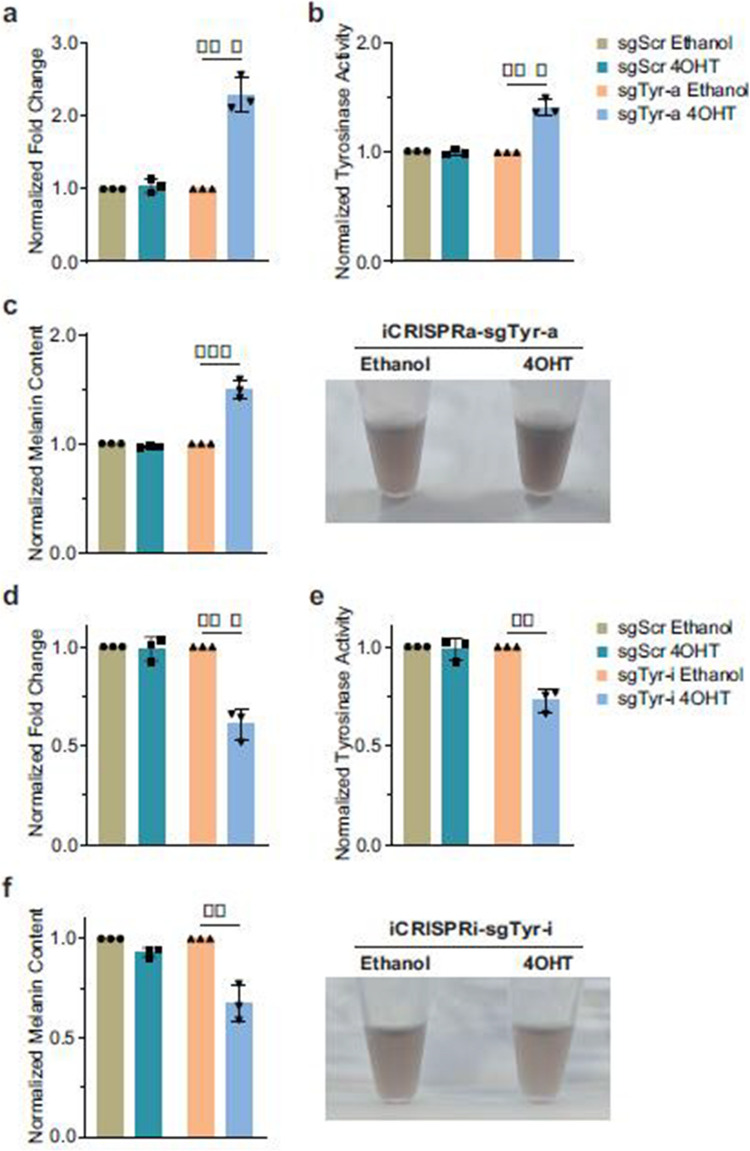


Corrected version
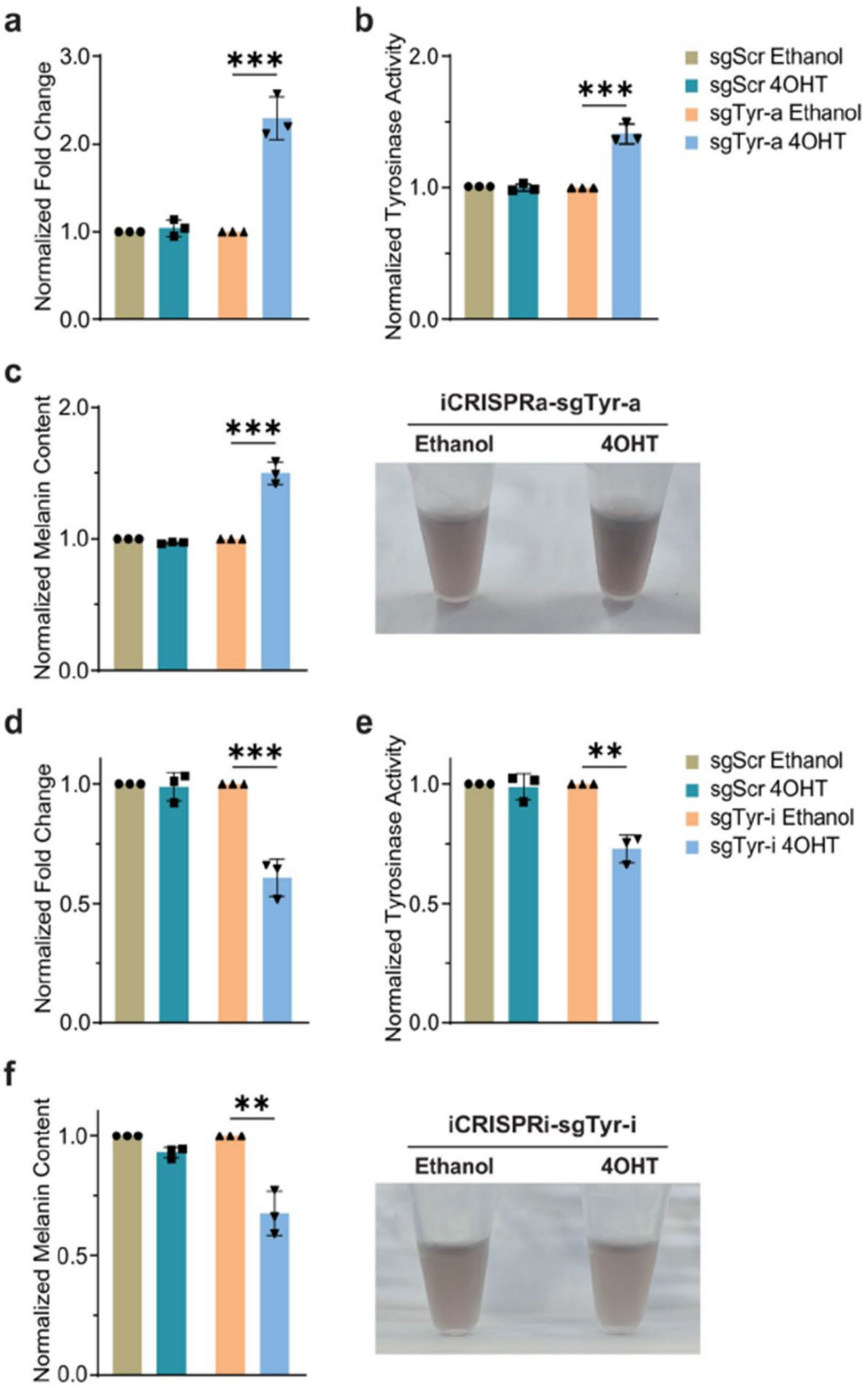


Fig 7

Incorrect version
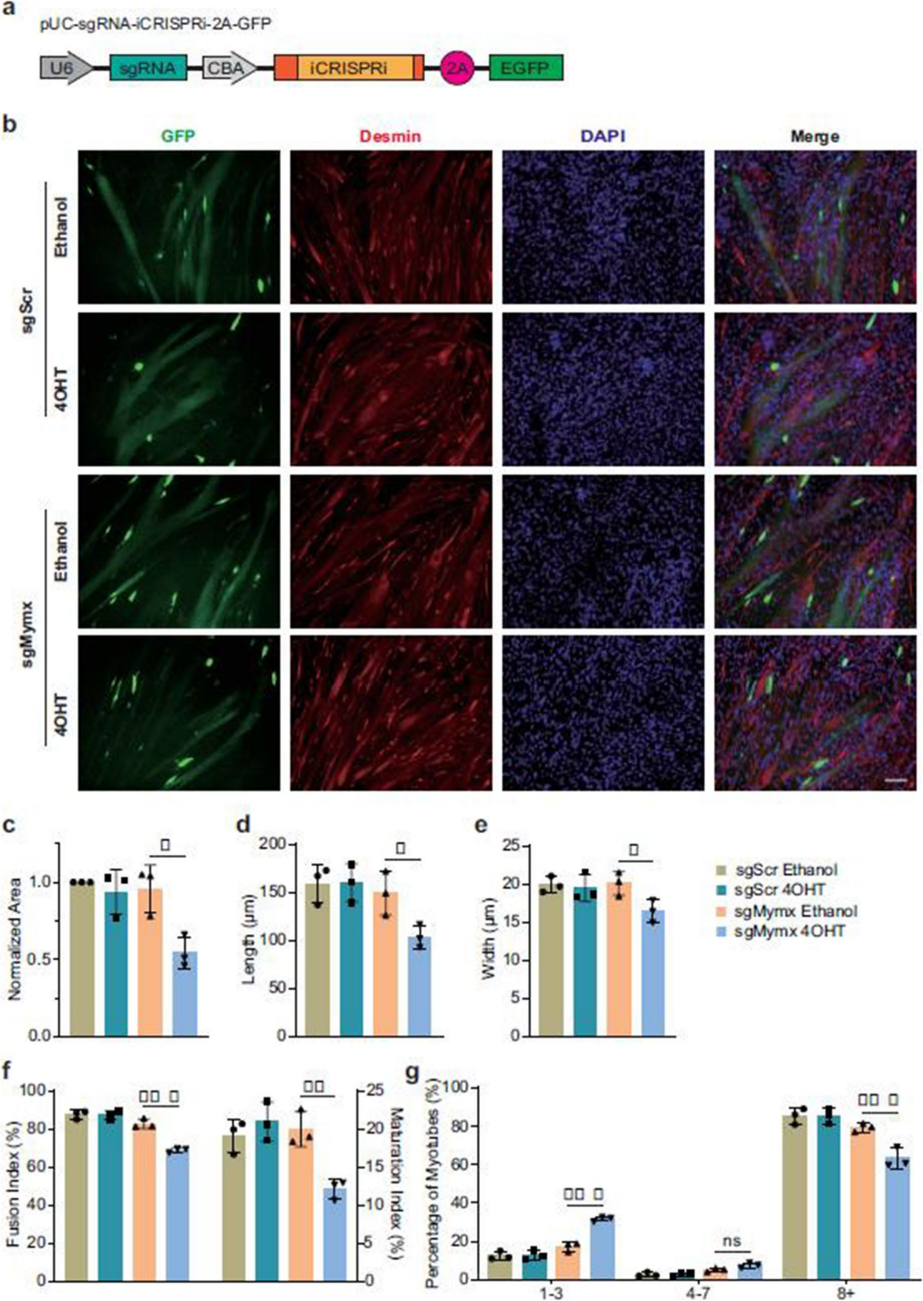


Corrected version
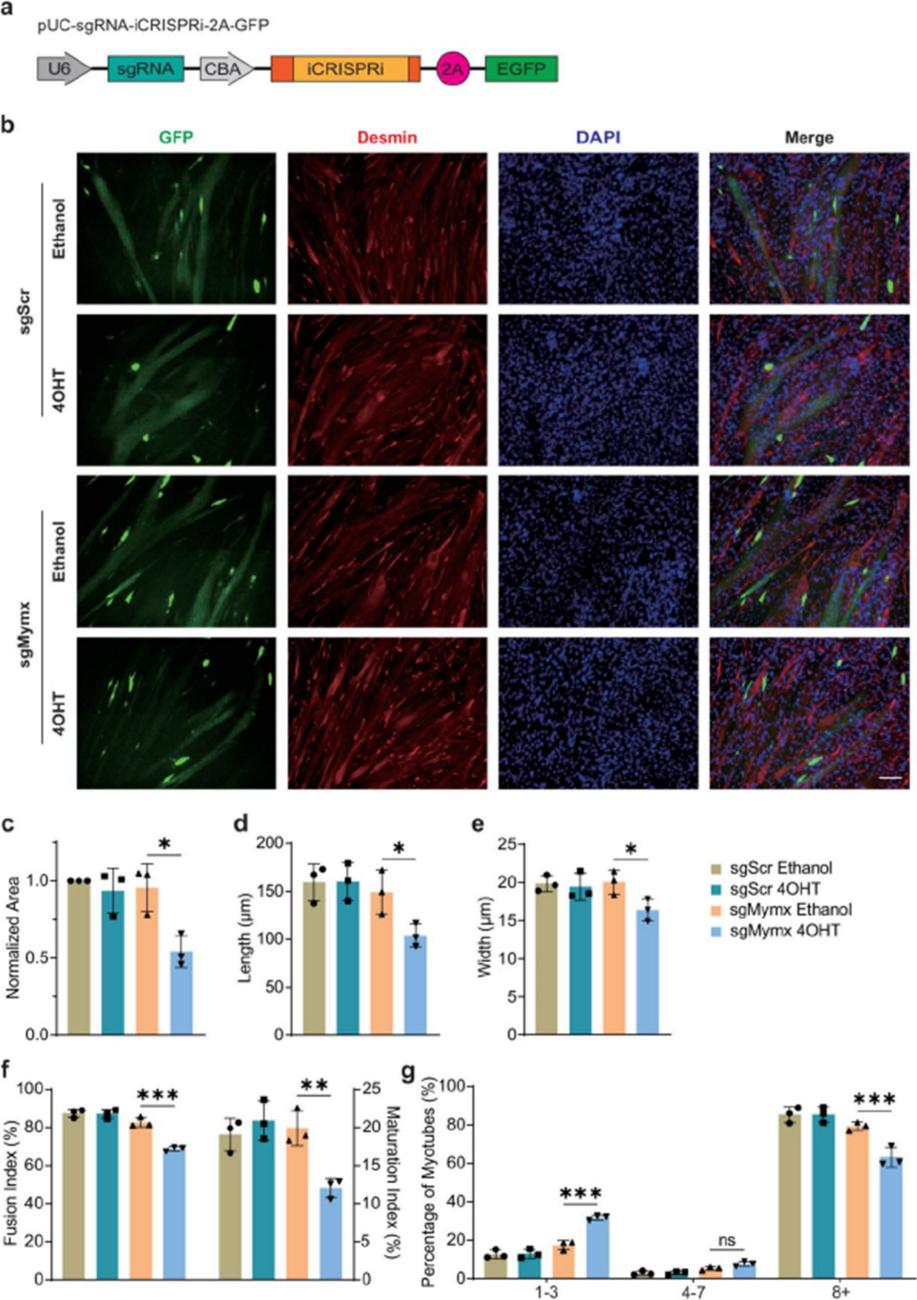


The original article has been corrected.

