# Supplementary figures and images for: Novel drug-inducible CRISPRa/i systems for rapid and reversible manipulation of gene transcription
Source: Cell Mol Life Sci. 2025 Jun 23;82(1):249. doi: 10.1007/s00018-025-05786-7 (PMC12185803; doi:10.1007/s00018-025-05786-7)

Fig. S1

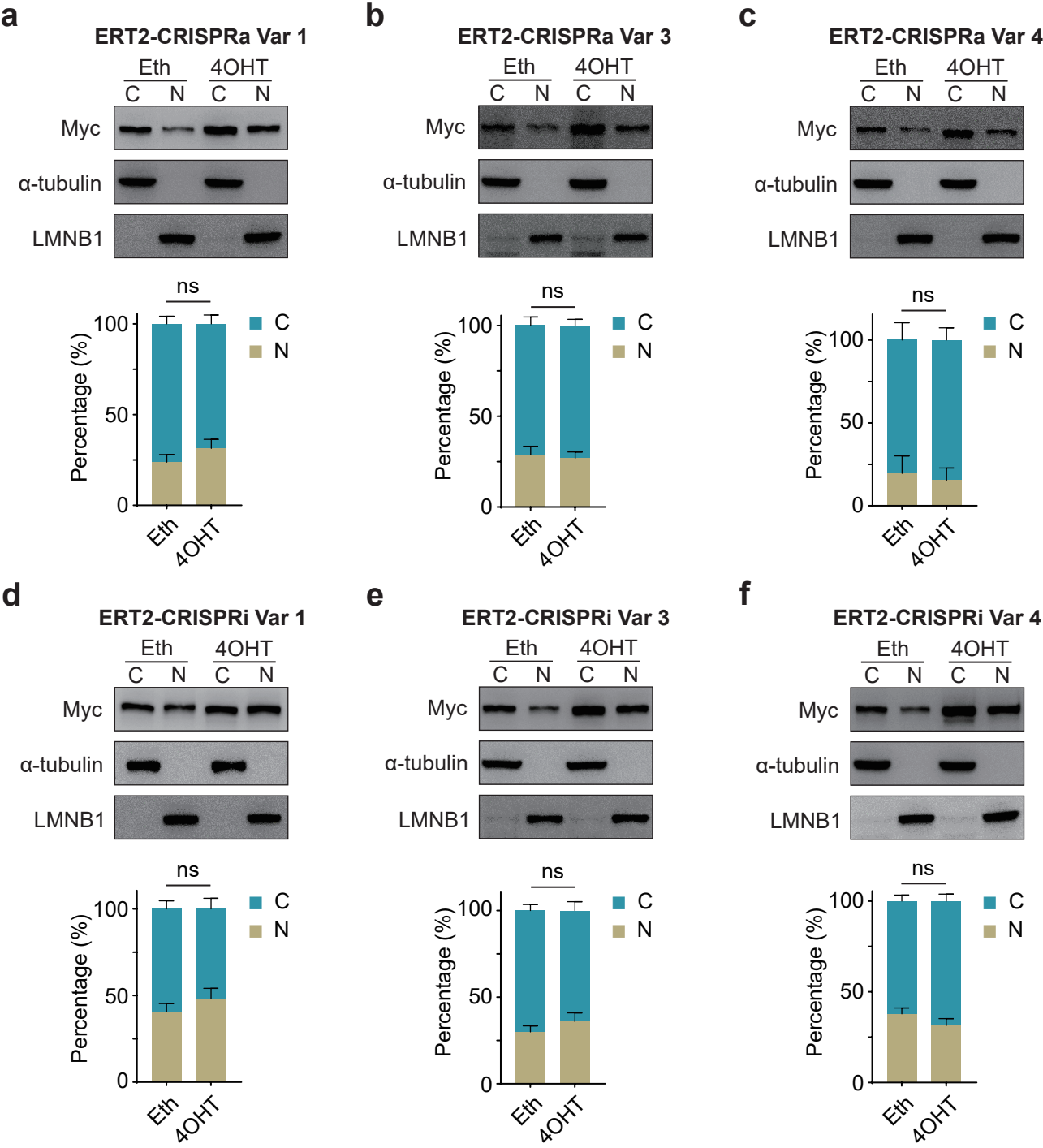

Fig. S2

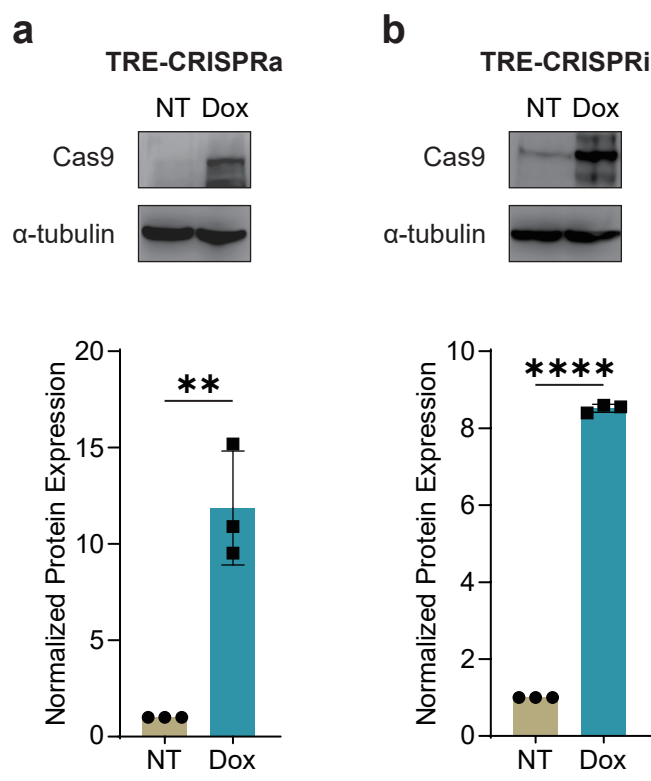

Fig. S3

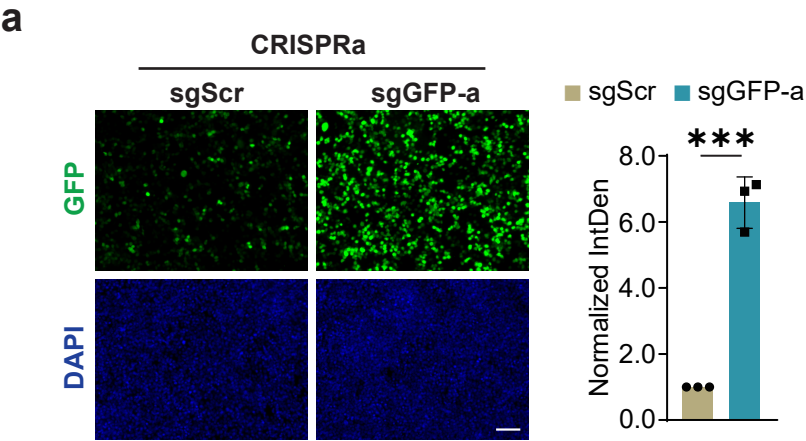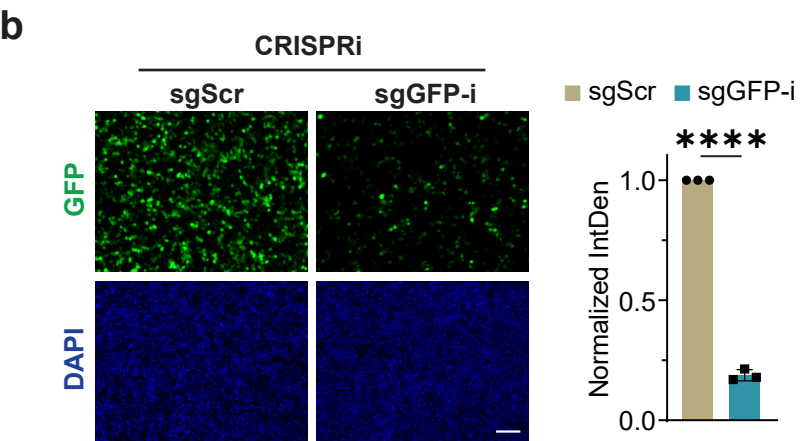

Fig. S4

a

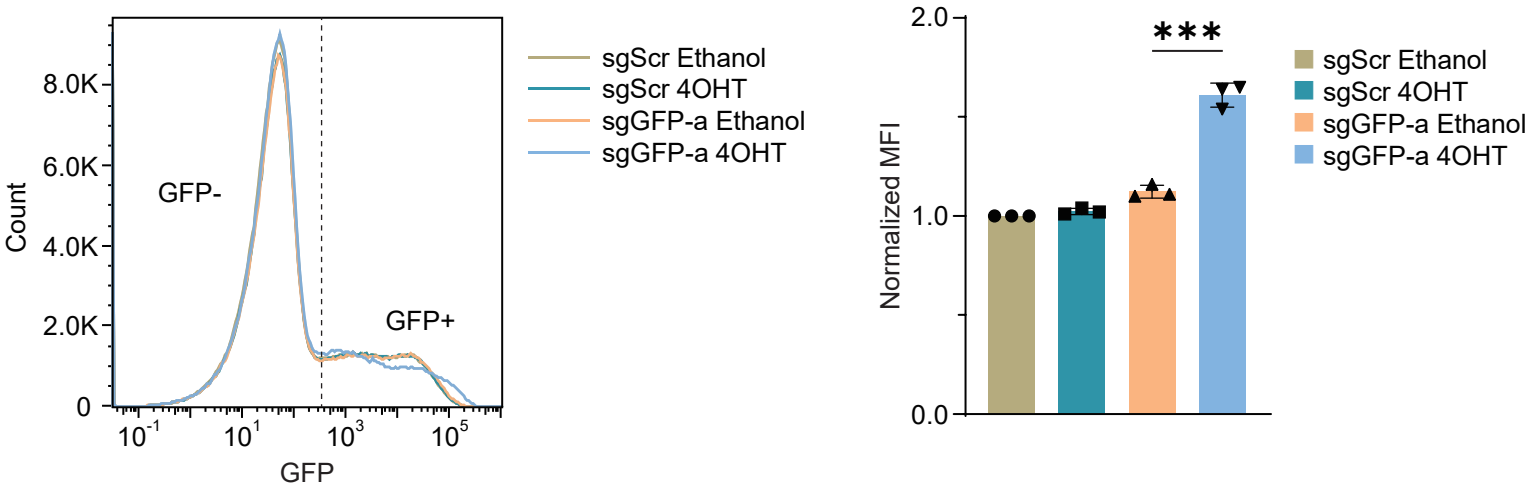

b

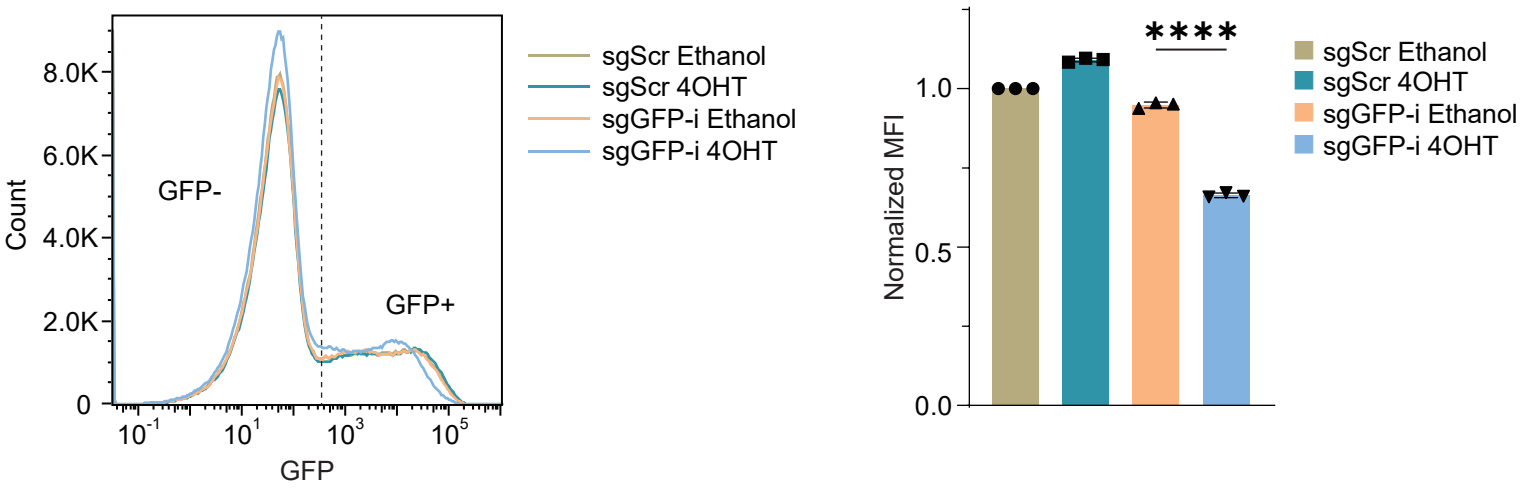

Fig. S5

a

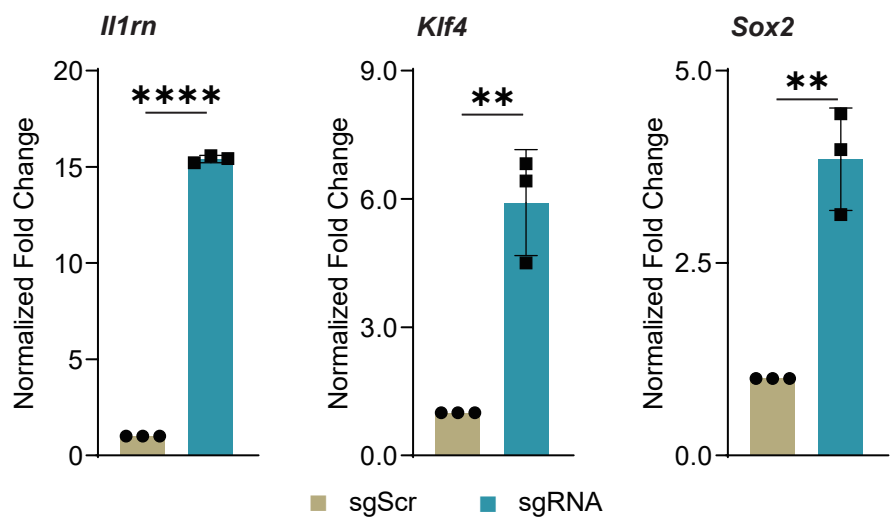

b

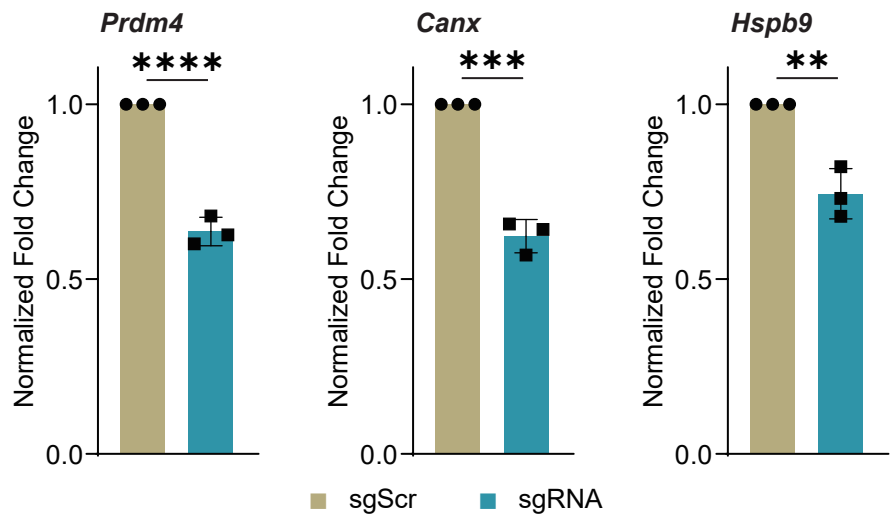

Fig. S6

a

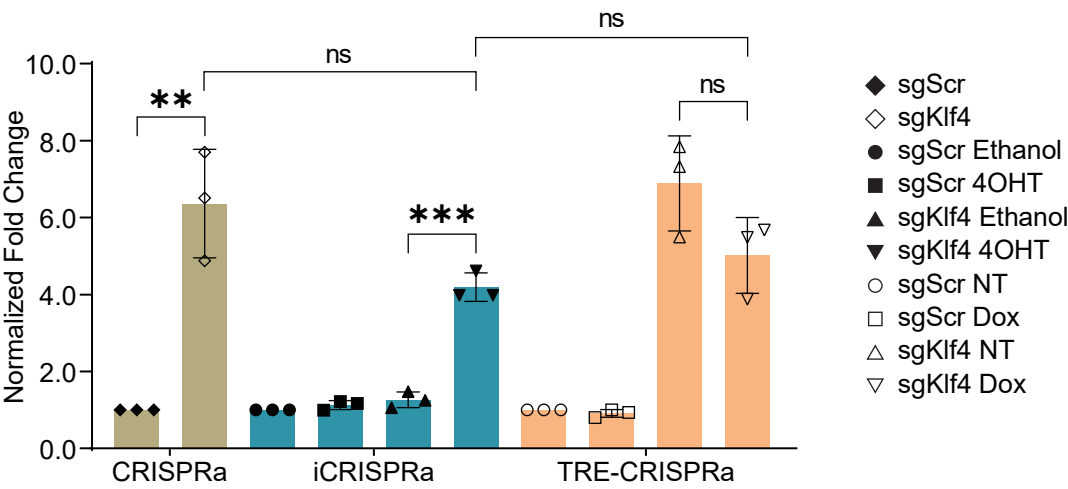

b

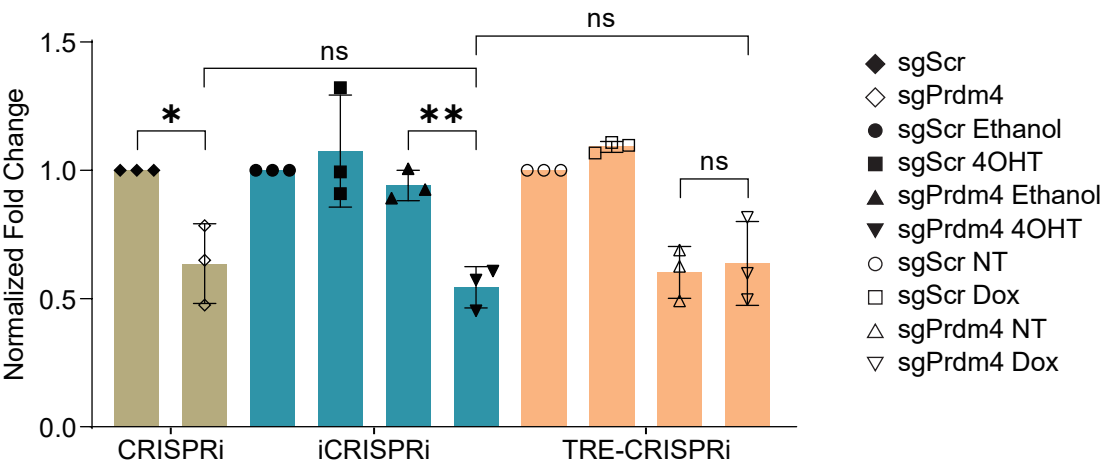

**Fig. S7**

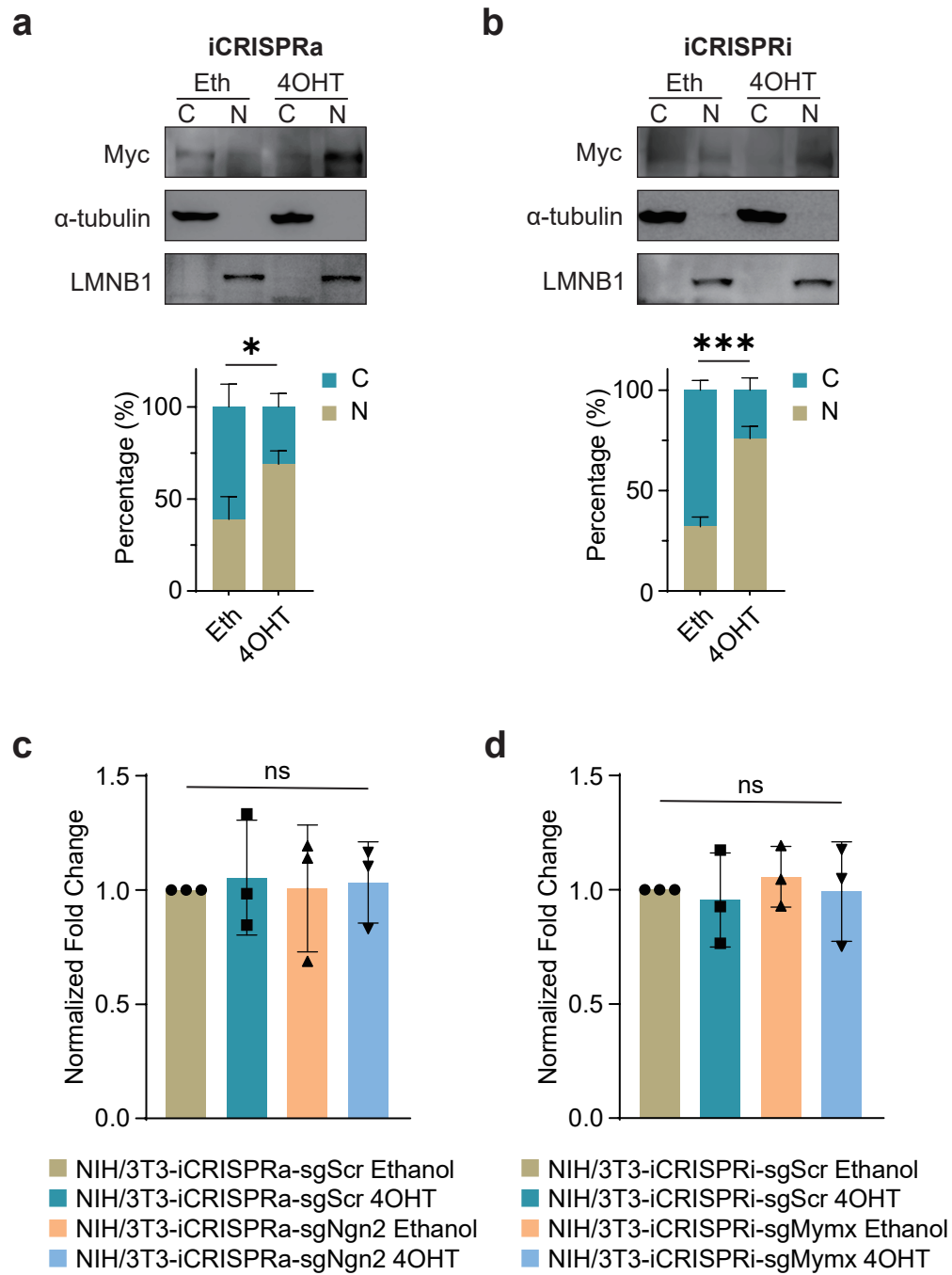

Fig. S8

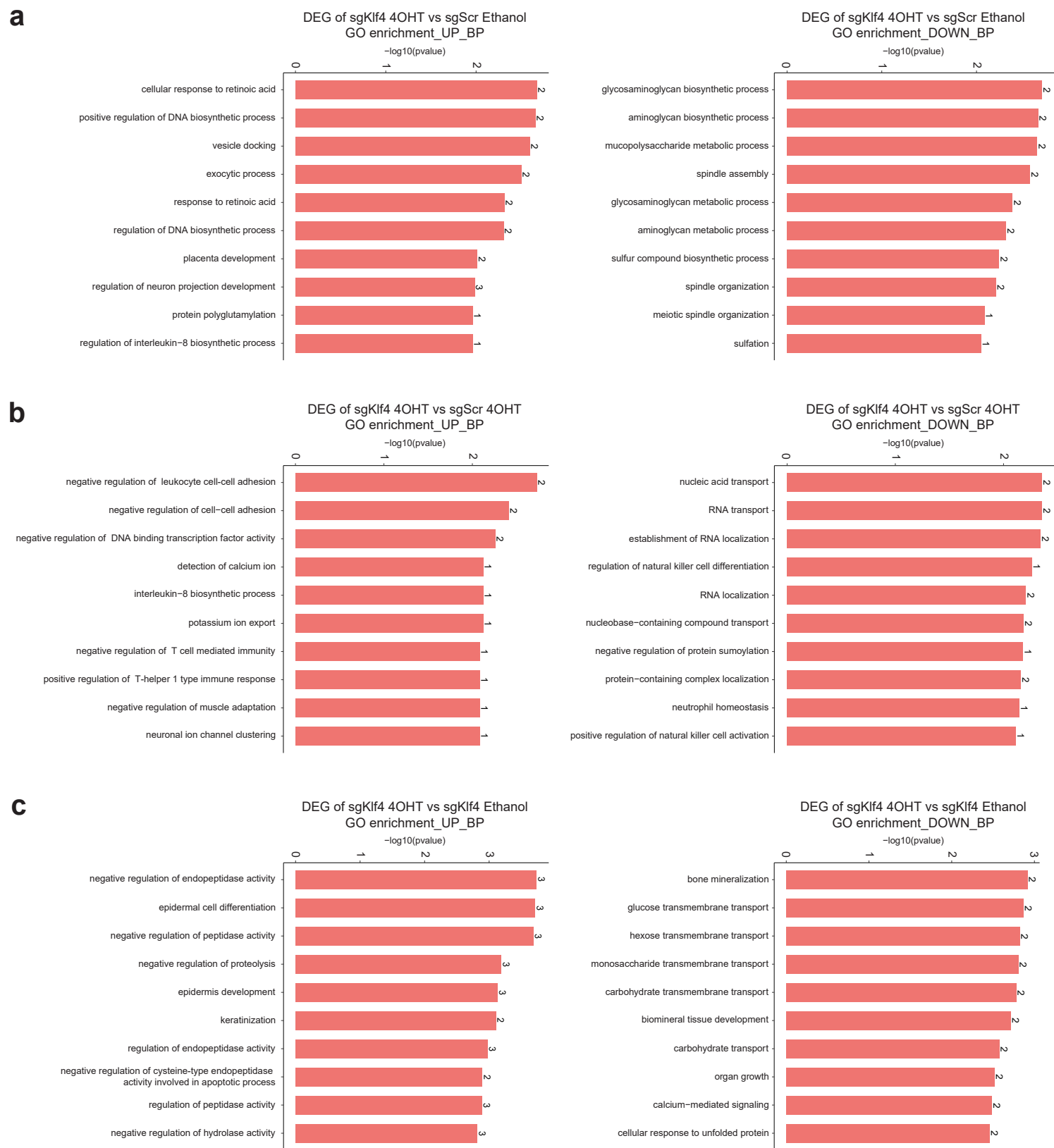

Fig. S9

a

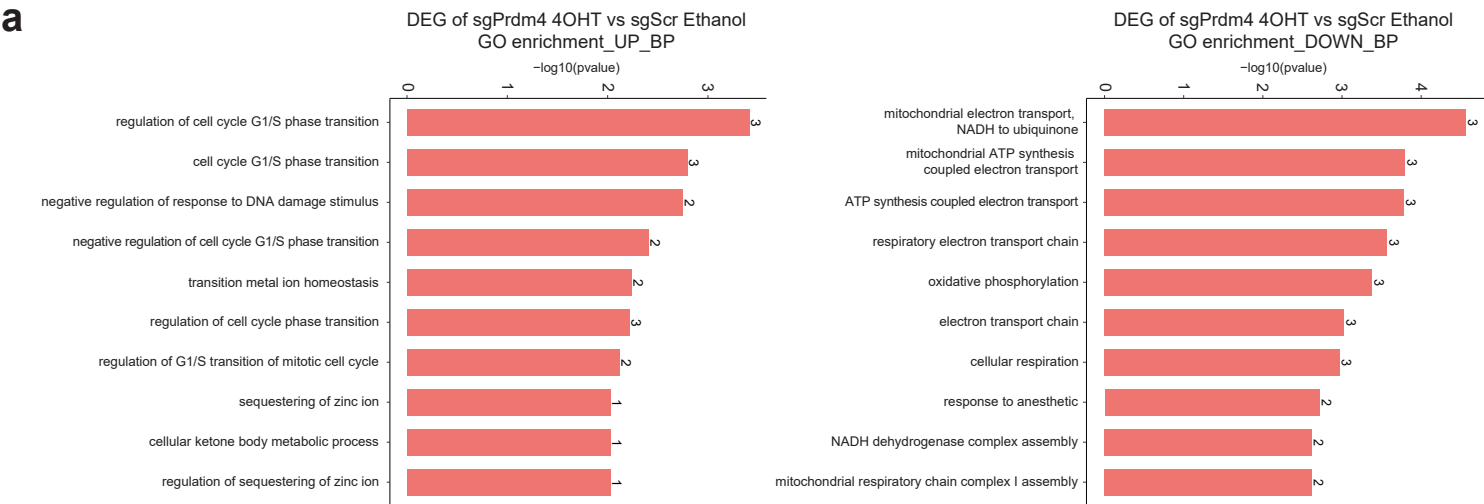

b

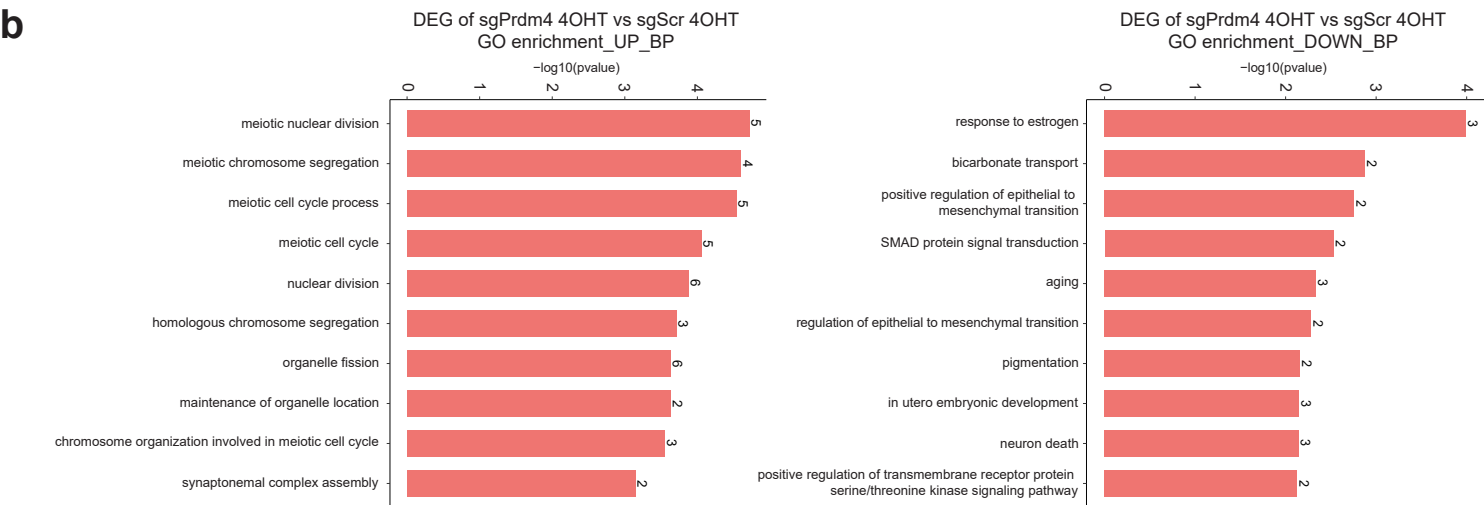

c

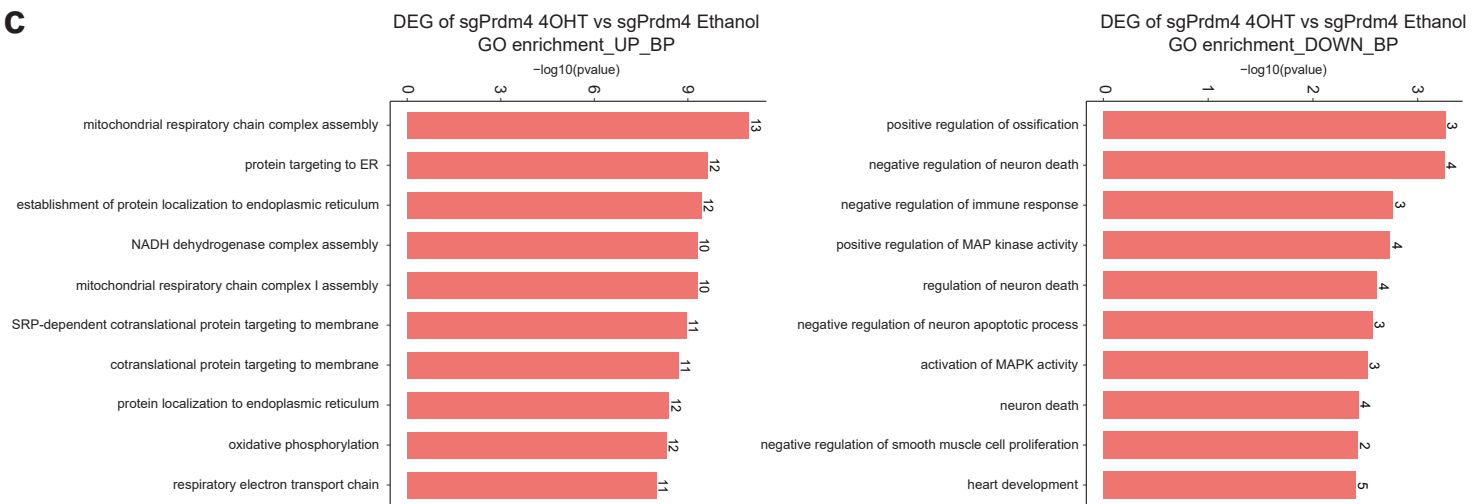

Fig. S10

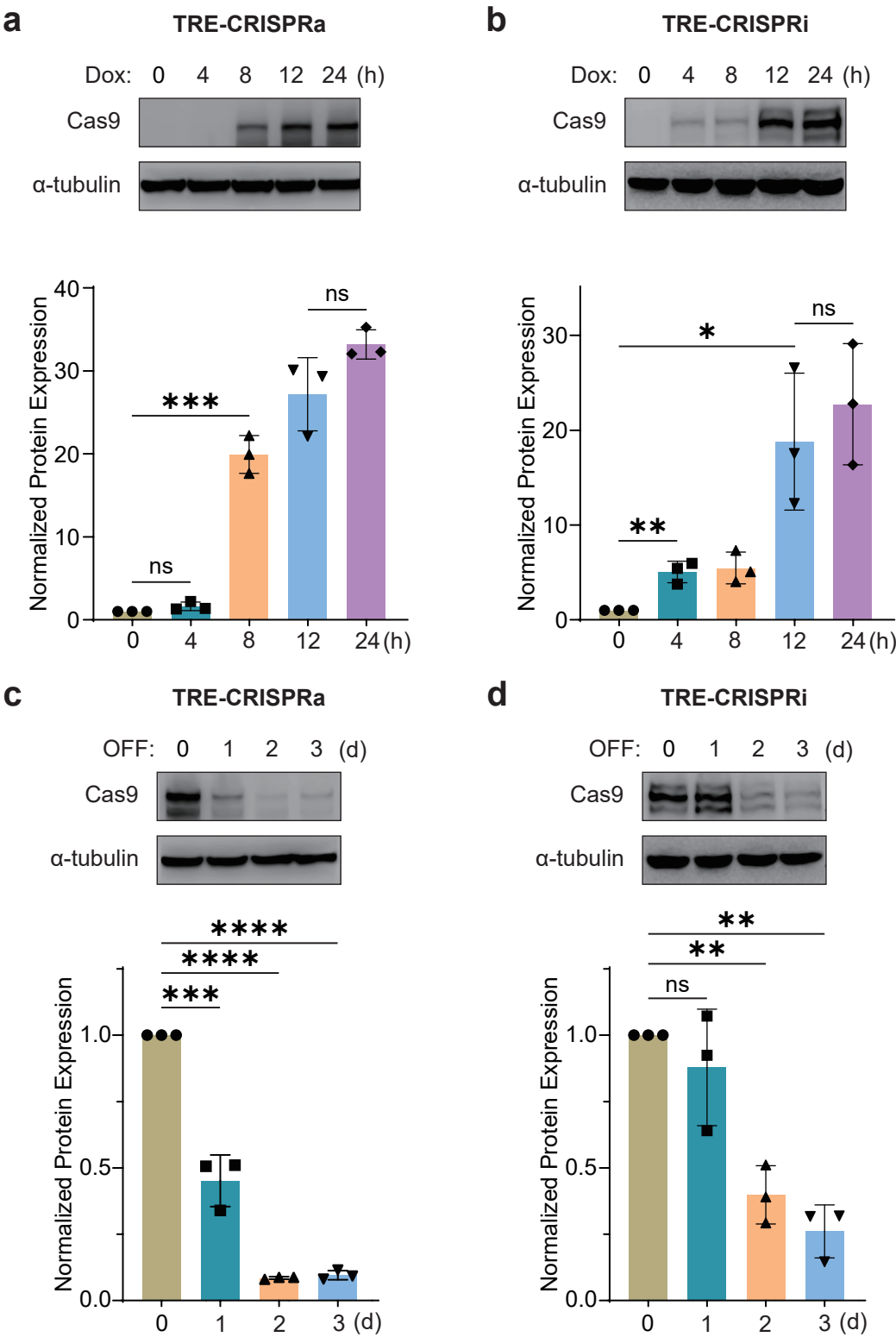

# Fig. S11

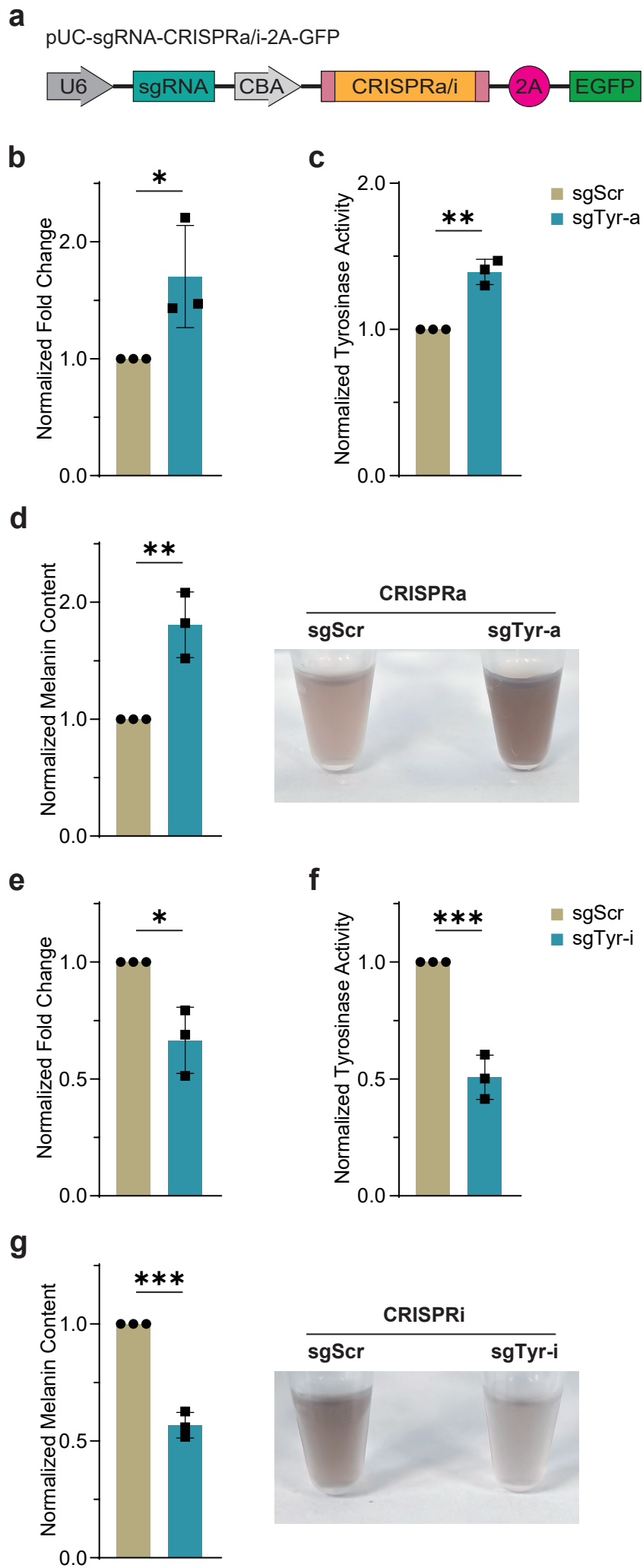

Fig. S12

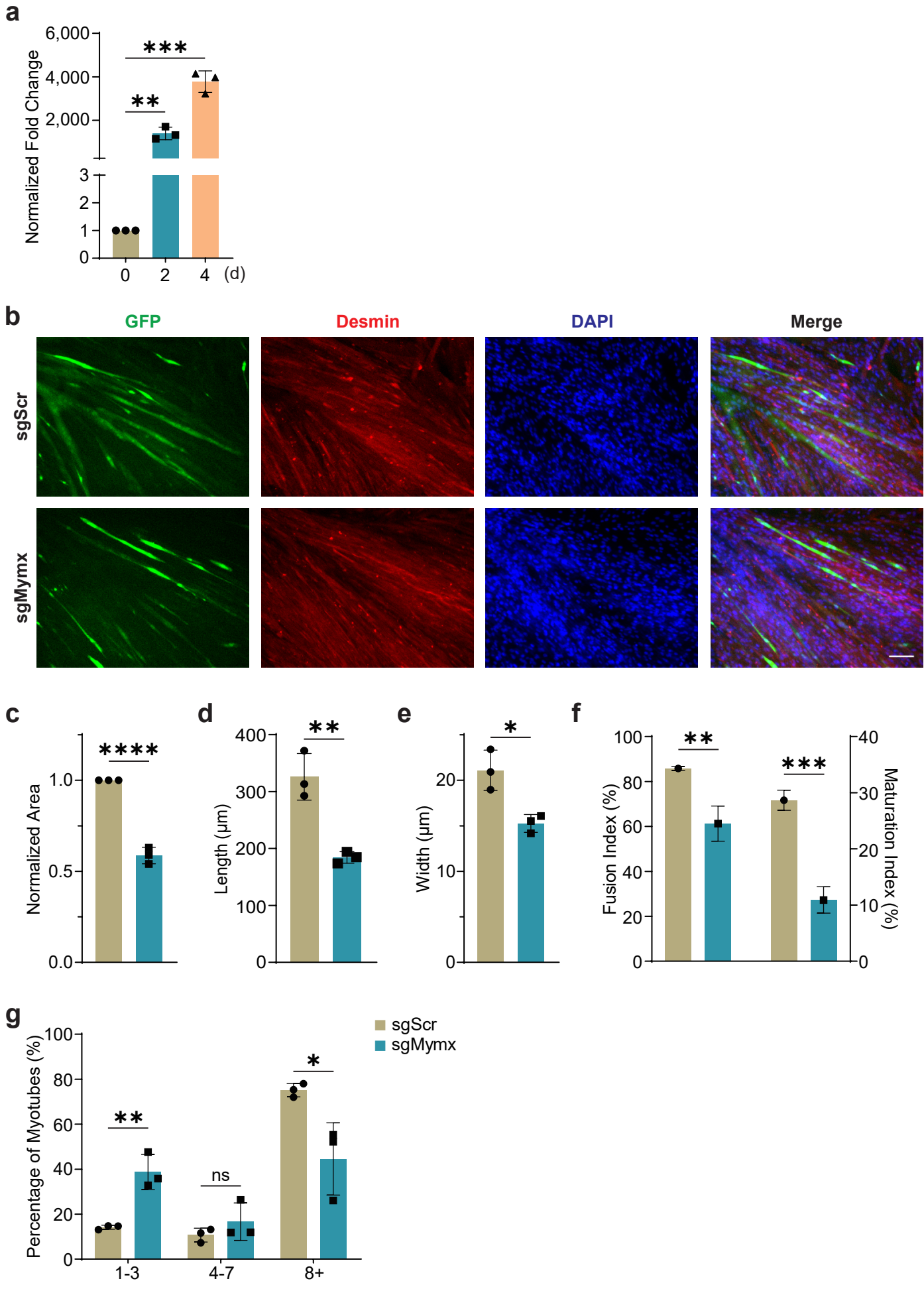

Supplement: Supplementary file 1 — Supplementary file1 Supplementary Fig. S1-12 (PDF 28.5 MB) [file 18_2025_5786_MOESM1_ESM.pdf]
