# Supplementary material for: Novel drug-inducible CRISPRa/i systems for rapid and reversible manipulation of gene transcription
Source: Cell Mol Life Sci. 2025 Jun 23;82(1):249. doi: 10.1007/s00018-025-05786-7 (PMC12185803; doi:10.1007/s00018-025-05786-7)
Supplement: Supplementary file 2 — Supplementary file2 Tables S1-3 and Supplementary Figure Legends (DOCX 36.8 KB) [file 18_2025_5786_MOESM2_ESM.docx]

Novel drug-inducible CRISPRa/i systems for rapid and reversible manipulation of gene transcription

*Cellular and Molecular Life Sciences*

Ming Sui, Meiling Zhou, Mengge Cui, Huan Liu, Xiaolin Zhang, Na Hu, Yang Li, Beibei Wang, Guojun Yang, Pengling Gui, Lingqiang Zhu, Feng Wan^*^ and Bin Zhang^*^

^*^ Corresponding authors: Bin Zhang, Department of Physiology, School of Basic Medicine, Tongji Medical College, Huazhong University of Science and Technology, Wuhan 430030, China, E-mail: binzhang@hust.edu.cn; Feng Wan, Department of Neurosurgery, Guangdong Provincial People's Hospital (Guangdong Academy of Medical Sciences), Southern Medical University, Guangzhou, 510080, China, E-mail: wanfeng@gdph.org.cn.

**Supplementary Data**

Tables S1-3 and Supplementary Figure Legends

**Table. S1** sgRNA sequences used in this study

| sgRNAs for transcriptional activation | |
| --- | --- |
| Target gene or site | sgRNA target sequence (5’-3’) |
| GFP | GCTCCACCCCATTGACGTCAA |
| Human *Il1rn* | CGGGGTTGGGGTAAGCACGA |
| Human *Klf4* | GCTGCCATAGCAACGATGGA |
| Human *Sox2* | GTGGCTGGCAGGCTGGCTCT |
| Mouse *Ngn2* | TGTGTTGTCGTTCTCGTGCG |
| Mouse *Isl1* | GGGAGTCCGATTTAAGCCGG |
| Mouse *Fgf7* | TTAGACAACGAACAGCTCAC |
| Mouse *Tyr* | TCAATTTAGTTACCTCACTA |
| sgRNAs for transcriptional repression | |
| Target gene or site | sgRNA target sequence (5’-3’) |
| GFP | GCTAACATGCGGTGACGTCG |
| Human *Prdm4* | GGGCGGGACGGGAAACGTTA |
| Human *Canx* | CTCCGCCTCTCTCTTTACTG |
| Human *Hspb9* | GTGGGACACCGGGATGCCACC |
| Mouse *Mstn* | GACTTTCTAATGCAAGCGGA |
| Mouse *Mymx* | CACCAGTTCTAACCGGCCAG |
| Mouse *Tyr* | AACATGTGATAGTCACTCCA |

**Table. S2** qPCR primers used in this study

| Gene | Species | Forward primer sequence (5’-3’) | Reverse primer sequence (5’-3’) |
| --- | --- | --- | --- |
| *Gapdh* | Human | CAAAGTTGTCATGGATGACC | CCATGGAGAAGGCTGGGG |
| *Il1rn* | Human | TTGCAAGGACCAAATGTCAATT | CTCATCACCAGACTTGACAC |
| *Sox2* | Human | TACAGCATGTCCTACTCGCAG | GAGGAAGAGGTAACCACAGGG |
| *Klf4* | Human | ACCAGGCACTACCGTAAACACA | GGTCCGACCTGGAAAATGCT |
| *Canx* | Human | GATCCAGACGCAGAGAAACC | CATCCAGGAGCTGACTCACA |
| *Prdm4* | Human | TCCTCTGTGAGCAATGCCTTG | CCACACATCACCCCTCGAT |
| *Hspb9* | Human | ACCATGCCAGAGACGGTTTC | CATGCGGTAACTGACCCTTTC |
| *Ctsw* | Human | AGATCCAGTTCAACCGGAGTT | TGTGAGGTCACTGAATGGAGT |
| *Znf419* | Human | ACCTTTGAGGATGTGGCTGT | TCACATTGCGGTAGAGGAGC |
| *Plk3* | Human | TTTTCGCACCACTTTGAGGAC | GAGGCCAGAAAGGATCTGCC |
| *Rhbdd1* | Human | GCCTATGTTATCACCGCATTTTC | GCTCCTTTTGAAGTCAGGTTCAT |
| *Ush1c* | Human | TCCATCAAAGTGAGACACATCG | CCGACACAAACTGATCCACATA |
| *Fgf20* | Human | GGGACTGGTCAGTATTAGAGGT | CTCTTGGAGTTCCGTCTTTGTT |
| *Nudt8* | Human | CTCTGCTCAGTGCGTGGG | CTTGTGCCTCCCGGTCAG |
| *Tmem106c* | Human | TTCACCGGGAGAGATAGCATC | AAGGACTGAATGCGGAAACAG |
| *Cpne7* | Human | GACCTCTTCAGCAAGTCCGAC | CACACCGGGTTCAGGTTGT |
| *Gart* | Human | ACCCGGTGTCGGTTTCATTT | TTTCCAGGCCAGCGTATGTT |
| *Gapdh* | Mouse | TGGCCTTCCGTGTTCCTAC | GAGTTGCTGTTGAAGTCGCA |
| *Isl1* | Mouse | CGGAAGAAACCAGCCTCAGT | GGGCATGCTTAAGAGACCCA |
| *Ngn2* | Mouse | CCGGACTCCACTTCACAGAG | GTCTTACGAGGTTCCCCACG |
| *Fgf7* | Mouse | AAGACTGTTCTGTCGCACCC | TTCCACCCCTTTGATTGCCA |
| *Mstn* | Mouse | AGTGGATCTAAATGAGGGCAGT | GGAGTACCTCGTGTTTTGTCTC |
| *Mymx* | Mouse | TTGATTCTGAGCAGTTCTGACTGG | CTTCTTGCCCTCCTGCTCAC |
| *Tyr* | Mouse | CTCTGGGCTTAGCAGTAGGC | GCAAGCTGTGGTAGTCGTCT |
| sgRNA | / | CCCTTGGAGAACCACCTTGT | CCGACTCGGTGCCACTTTTT |

**Table. S3** On-target and non-specific off-target sites investigated in this study

| sgRNA | On- or off-target | Gene name | Genomic locus | Sequence (5’-3’) ^*^ |
| --- | --- | --- | --- | --- |
| Il1rn | On-target | *Il1rn* | chr2: 113760244-113990243 | CGGGGTTGGGGTAAGCACGA |
|  | Off-target 1 | *Ctsw* | chr11: 65635749-65658748 | tGGGGcTGGaGaAAcCACGA |
|  | Off-target 2 | *Prdm4* | chr12: 108153710-108156009 | CGGGGagGGcGcAcGCACGA |
| Klf4 | On-target | *Klf4* | chr9: 110240671-110263670 | GCTGCCATAGCAACGATGGA |
|  | Off-target 1 | *Znf419* | chr19: 57986513-58009512 | aaTGCCATAGCtgCcATGGA |
|  | Off-target 2 | *Plk3* | chr1: 45265520-45267819 | GgTGgCATgGgAACcATGGA |
| Sox2 | On-target | *Sox2* | chr3: 181428368-181430667 | GTGGCTGGCAGGCTGGCTCT |
|  | Off-target 1 | *Hspb9* | chr17: 40273529-40275828 | GgcGCTGaCAGtCTGGCTCT |
|  | Off-target 2 | *Rhbdd1* | chr2: 227596683-227803682 | tTGcCTtGCtGGCTGGCTCT |
| Prdm4 | On-target | *Prdm4* | chr12: 108153710-108156009 | GGGCGGGACGGGAAACGTTA |
|  | Off-target 1 | *Tmem106c* | chr12: 48356456-48358755 | GaGaGGaAtGGGAAtCGTTA |
|  | Off-target 2 | *Nudt8* | chr11: 67396520-67398819 | cGGtGGGACGGGgtcCGTTA |
| Canx | On-target | *Canx* | chr5: 179124878-179127177 | CTCCGCCTCTCTCTTTACTG |
|  | Off-target 1 | *Ush1c* | chr11: 17564498-17566797 | tTCCctCcCTCaCTTTACTG |
|  | Off-target 2 | *Fgf20* | chr8: 16858433-16860732 | CcCCaCaaCaCTCTTTACTG |
| Hspb9 | On-target | *Hspb9* | chr17: 40273765-40276064 | TGGGACACCGGGATGCCACC |
|  | Off-target 1 | *Cpne7* | chr16: 89641355-89643654 | TGGGAggCCGaGAgGCCACC |
|  | Off-target 2 | *Gart* | chr21: 34913230-34915529 | accGACACCGGGtgGCCACC |

^*^The lowercase letters in the sequences indicate the mismatched nucleotide, meaning they represent the nucleotides in the off-target sequences that do not match their intended targets.

**Figure Legends**

**Fig. S1** Western blot and quantification of subcellular localization of ERT2-CRISPRa/i variants. HEK 293T cells transfected with ERT2-CRISPRa (**a-c**) and ERT2-CRISPRi (**d-f**) variants were separated into cytoplasmic and nuclear fractions after ethanol or 4OHT treatment for 24 h. ERT2-CRISPRa/i variants tagged with Myc tags could be detected with anti-Myc antibodies. α-tubulin served as a cytoplasmic marker, while LMNB1 served as a nuclear marker. Var: variant. Eth: ethanol. C: cytoplasmic fraction, N: nuclear fraction. Data are presented as mean ± SD, n = 3 biological replicates. ns, not significant (*p* > 0.05); Student’s *t*-test.

**Fig. S2** Western blot and quantification of Dox-inducible TRE-CRISPRa/i proteins. HEK 293T cells were transfected with TRE-CRISPRa (**a**) and TRE-CRISPRi (**b**) plasmids and non-treated or treated with 1 µg/mL doxycycline for 24 h. The total proteins were extracted, and CRISPRa/i proteins were detected with anti-Cas9 antibodies by Western blot. α-tubulin served as a loading control. NT: non-treated. Dox: doxycycline. Data are presented as mean ± SD, n = 3 biological replicates. ** *p* < 0.01, **** *p* < 0.0001; Student’s *t*-test.

**Fig. S3** Exogenous GFP transcriptional activation and repression assays. HEK 293T cells were co-transfected with GFP, sgGFP-a/i targeting CMV promoter region of GFP and CRISPRa (**a**) or CRISPRi (**b**) plasmids and cultured for 48 h. The integrated densities of GFP signal were calculated by ImageJ and normalized to DAPI. IntDen: integrated density. Scale bars, 100 μm. All photographs shown in this figure are representative of three independent experiments. Each data point represents an average of multiple fields of one experiment sample. Data are presented as mean ± SD, n = 3 biological replicates. *** *p* < 0.001, **** *p* < 0.0001; Student’s *t*-test.

**Fig. S4** GFP transcriptional regulation detected by flow cytometry. HEK 293T cells were co-transfected with GFP reporter driven by CMV promoter, sgGFP-a/i targeting CMV promoter region of GFP and iCRISPRa (**a**) or iCRISPRi (**b**) plasmids and treated with either ethanol or 4OHT for 48 h. Approximately 100,000 GFP-positive cells were counted for each sample and the mean fluorescence intensity of GFP was analyzed using FlowJo software. MFI: mean fluorescence intensity. Graphs are representative data sets from one of three independent experiments. Data are presented as mean ± SD, n = 3 biological replicates. *** *p* < 0.001, **** *p* < 0.0001; Student’s *t*-test.

**Fig. S5** Detection of transcriptional regulation of endogenous genes by CRISPRa/i. (**a**) *Il1rn*, *Klf4* and *Sox2* transcripts were targeted with the CRISPRa system to activate transcription, (**b**) *Prdm4*, *Canx* and *Hspb9* transcripts were targeted with the CRISPRi system to repress transcription. Transcriptional levels of target genes of each sample were detected by qPCR and normalized to the housekeeping gene *Gapdh*. Data are presented as mean ± SD, n = 3 biological replicates. ** *p* < 0.01, *** *p* < 0.001, **** *p* < 0.0001; Student’s *t*-test.

**Fig. S6** Benchmarking iCRISPRa/i systems to non-inducible and Dox-inducible CRISPRa/i systems. iCRISPRa/i, non-inducible and Dox-inducible CRISPRa/i systems and targeting sgRNA were transfected into HEK 293T cells and treated with their inducers to activate (**a**) or repress (**b**) target transcripts. Transcriptional levels of target genes of each sample were detected by qPCR and normalized to the housekeeping gene *Gapdh*. NT: non-treated. Dox: doxycycline. Data are presented as mean ± SD, n = 3 biological replicates. ns, not significant (*p* > 0.05), * *p* < 0.05, ** *p* < 0.01, *** *p* < 0.001; Student’s *t*-test.

**Fig. S7** Subcellular localization of iCRISPRa/i proteins and transcriptional levels of sgRNAs in stable NIH/3T3 cells. (**a, b**) NIH/3T3-iCRISPRa/i cells were separated into cytoplasmic and nuclear fractions after ethanol or 4OHT treatment for 2 d. iCRISPRa/i proteins tagged with Myc tags were detected by anti-Myc antibodies. α-tubulin served as a cytoplasmic marker, while LMNB1 served as a nuclear marker. Eth: ethanol. C: cytoplasmic fraction, N: nuclear fraction. * *p* < 0.05, *** *p* < 0.001; Student’s *t*-test. (**c, d**) The transcriptional levels of control and target sgRNAs were detected by qPCR after ethanol or 4OHT treatment for 2 d in NIH/3T3-iCRISPRa/i-sgRNA cells. ns, not significant (*p* > 0.05); two-way ANOVA. Data are presented as mean ± SD, n = 3 biological replicates.

**Fig. S8** GO enrichment analysis of the up/downregulated DEGs found in iCRISPRa system. (**a**) GO categories of BP in the DESs between sgKlf4 4OHT and sgScr Ethanol. (**b**) GO categories of BP in the DESs between sgKlf4 4OHT and sgScr 4OHT. (**c**) GO categories of BP in the DESs between sgKlf4 4OHT and sgPrdm4 Ethanol.

**Fig. S9** GO enrichment analysis of the up/downregulated DEGs found in iCRISPRi system. (**a**) GO categories of BP in the DESs between sgPrdm4 4OHT and sgScr Ethanol. (**b**) GO categories of BP in the DESs between sgPrdm4 4OHT and sgScr 4OHT. (**c**) GO categories of BP in the DESs between sgPrdm4 4OHT and sgPrdm4 Ethanol.

**Fig. S10** Expression pattern kinetics of TRE-CRISPRa/i systems. (**a, b**) Protein expression patterns of TRE-CRISPRa (**a**) and TRE-CRISPRi (**b**) with doxycycline treatment for 0 h, 4 h, 8 h, 12 h and 24 h were detected and quantified. (**c, d**) Protein expression patterns of TRE-CRISPRa (**c**) and TRE-CRISPRi (**d**) after doxycycline retraction. Transfected HEK 293T cells were treated with doxycycline for 24 h. After induction, doxycycline was withdrawn and the protein expression levels were detected at 0 d, 1 d, 2 d and 3 d. TRE-CRISPRa/i proteins were detected with anti-Cas9 antibodies by Western blot. α-tubulin served as a loading control. Dox: doxycycline. Data are presented as mean ± SD, n = 3 biological replicates. ns, not significant (*p* > 0.05), * *p* < 0.05, ** *p* < 0.01, *** *p* < 0.001, **** *p* < 0.0001; Student’s *t*-test.

**Fig. S11** CRISPRa/i changed tyrosinase activity and melanin content by altering tyrosinase gene transcription. (**a**) Schematic of pUC-sgRNA-CRISPRa/i-2A-GFP. sgRNA was driven by the U6 promoter, CRISPRa/i and GFP were driven by the Chicken β-actin (CBA) promoter and split by a self-cleaving 2A peptide. (**b-d**) The *Tyr* transcript (**b**), tyrosinase activity (**c**) and melanin content (**d**) of CRISPRa-transfected cells were analyzed. (**e-g**) The *Tyr* transcript (**e**), tyrosinase activity (**f**) and melanin content (**g**) of CRISPRi-transfected cells were analyzed. Transcriptional levels of *Tyr* of each sample were detected by qPCR and normalized to the housekeeping gene *Gapdh*. Tyrosinase activities were determined by L-DOPA oxidation. Melanin contents were measured by a Mexameter. Data are presented as mean ± SD, n = 3 biological replicates. * *p* < 0.05, ** *p* < 0.01, *** *p* < 0.001; Student’s *t*-test.

**Fig. S12** Repressing *Mymx* transcription by CRISPRi caused the reduction of C2C12 cell fusion. (**a**) The transcriptional levels of *Mymx* during C2C12 myoblasts differentiation at fusion Day 0, Day 2 and Day 4. Transcriptional levels of *Mymx* of each sample were detected by qPCR and normalized to the housekeeping gene *Gapdh*. (**b**) Immunofluorescence images of C2C12 cells that fusion of myoblasts was inhibited by iCRISPRi. Transfected cells were differentiated, fixed and stained with antibodies against Desmin and cell nuclei were stained with DAPI. Scale bar, 100 μm. All photographs were representative of three independent experiments. (**c-e**) Quantification of the normalized area (**c**), length (**d**) and width (**e**) of GFP-positive cells. (**f, g**) Quantification of the fusion and maturation index (**f**) and the percentage of cells with 1–3, 4–7 and 8+ nuclei (**g**). Data are presented as mean ± SD, n = 3 biological replicates. ns, not significant (*p* > 0.05), * *p* < 0.05, ** *p* < 0.01, *** *p* < 0.001, **** *p* < 0.0001; Student’s *t*-test.
